# Stable Isotope Turnover and Half-Life in Animal Tissues: A Literature Synthesis

**DOI:** 10.1371/journal.pone.0116182

**Published:** 2015-01-30

**Authors:** M. Jake Vander Zanden, Murray K. Clayton, Eric K. Moody, Christopher T. Solomon, Brian C. Weidel

**Affiliations:** 1 Center for Limnology, University of Wisconsin—Madison, Madison, Wisconsin, United States of America; 2 Department of Statistics, University of Wisconsin—Madison, Madison, Wisconsin, United States of America; 3 School of Life Sciences, Arizona State University, Tempe, Arizona, United States of America; 4 Department of Natural Resource Sciences, McGill University, Ste. Anne de Bellevue, Quebec, Canada; 5 Lake Ontario Biological Station, USGS, Oswego, New York, United States of America; Scottish Association for Marine Science, UNITED KINGDOM

## Abstract

Stable isotopes of carbon, nitrogen, and sulfur are used as ecological tracers for a variety of applications, such as studies of animal migrations, energy sources, and food web pathways. Yet uncertainty relating to the time period integrated by isotopic measurement of animal tissues can confound the interpretation of isotopic data. There have been a large number of experimental isotopic diet shift studies aimed at quantifying animal tissue isotopic turnover rate λ (%·day^-1^, often expressed as isotopic half-life, ln(2)/λ, days). Yet no studies have evaluated or summarized the many individual half-life estimates in an effort to both seek broad-scale patterns and characterize the degree of variability. Here, we collect previously published half-life estimates, examine how half-life is related to body size, and test for tissue- and taxa-varying allometric relationships. Half-life generally increases with animal body mass, and is longer in muscle and blood compared to plasma and internal organs. Half-life was longest in ecotherms, followed by mammals, and finally birds. For ectotherms, different taxa-tissue combinations had similar allometric slopes that generally matched predictions of metabolic theory. Half-life for ectotherms can be approximated as: ln (half-life) = 0.22*ln (body mass) + group-specific intercept; n = 261, p<0.0001, r^2^ = 0.63. For endothermic groups, relationships with body mass were weak and model slopes and intercepts were heterogeneous. While isotopic half-life can be approximated using simple allometric relationships for some taxa and tissue types, there is also a high degree of unexplained variation in our models. Our study highlights several strong and general patterns, though accurate prediction of isotopic half-life from readily available variables such as animal body mass remains elusive.

## Introduction

Natural variability in the stable isotopic ratios of carbon, nitrogen, and sulfur (δ^13^C, δ^15^N, δ^34^S) are widely used in animal ecology, including studies of animal migration, food webs, trophic position estimation, and food source reliance [1–3]. The use of stable isotopes in food web studies is predicated on an understanding of how the isotopic composition of animal diet and tissues are related. There are two central aspects to this. First are diet-tissue discrimination factors (sometimes referred to as trophic fractionation). Patterns and variability in diet-tissue discrimination factors have been summarized [4–6]. The second relates to temporal isotopic dynamics—specifically the idea that an animal tissue does not immediately reflect the isotopic composition of it's diet, but rather integrates over some period of time. Notably, many stable isotope field studies such as those that use isotopic mixing models [7,8] tacitly assume that the isotopic composition of animal tissues is in equilibrium with diet (i.e., assumes diet-tissue steady state). This is clearly not the case in many situations, and can lead to highly misleading food web interpretations [9].

Fry and Arnold [10] was the first study to quantify the rate of isotopic incorporation in animal tissues. By shifting the carbon isotopic composition of the diets of laboratory-reared shrimp, they quantified the time-scale of isotopic incorporation and estimated isotopic turnover rate (λ, %·day^-1^) and isotopic half-life (ln(2)/λ, days), defined as the time required to reach 50% equilibration with the diet. Fry and Arnold also noted that isotopic turnover occurs as a result of two distinct processes: tissue growth and catabolic turnover. Subsequent laboratory studies have estimated isotopic turnover for a wide variety of animals and tissue types [1,11]. These studies have revealed tissue-specific differences, for example, internal organs and blood plasma tend to have high rates of isotopic incorporation compared to muscle tissue and blood cells [11]. Since tissues integrate consumer diets at different time scales, examination of multiple tissues can potentially provide information about the temporal dynamics of resource use [12].

Interpretation of the isotopic value of a tissue from a field study should consider, at least in a general sense, the rate of isotopic incorporation. How much do we currently know about the time scale of isotopic incorporation for different tissues and animals? Do rates of isotopic incorporation differ systematically among taxa, tissue types, or element (δ^13^C, δ^15^N, δ^34^S)? How variable is it, and to what extent can we make broad generalities in the absence of taxa- or system-specific isotopic incorporation information? Over the past several decades, a large number of isotopic diet shift experiments have estimated isotopic half-life for a diverse range of animal taxa and tissue types. A quantitative synthesis of these isotopic half-life results has recently been identified as a ‘fruitful, and perhaps urgent task’ in a recent review [1]. Several recent studies have summarized isotopic half-life for specific taxa and tissue types [13–16]. The most exhaustive synthesis to date [11] examined differences among tissue types for three major animal taxa (bird, mammal, fishes), but did not consider the role of body size. One would expect isotopic turnover to vary strongly with body size, with tissues of small animals integrating over a short time period relative to large animals. The basis for the role of body size was formalized by Carleton and Martinez del Rio [15], who noted that rates of protein turnover tend to be proportional to body mass to approximately the 3/4 power [17]. Since the mass of individual tissues is roughly proportional to body mass, isotopic turnover would be expected to be roughly proportional to body mass^-1/4^ (i.e., mass^3/4^/mass). Allometric studies of isotopic half-life for specific taxa and tissue types have generally been consistent with this predicted slope of ~ 0.25 [14,15]. In this study, we assemble an extensive collection of published estimates of isotopic half-life and test whether half-life varies as a function of body mass, as predicted from theory [1,18]. We also test whether there are differences in allometic relationships among taxa, tissue types, and three widely used isotopes (δ^13^C, δ^15^N and δ^34^S). A general understanding of how isotopic half-life varies with body mass for different tissue types and taxa is urgently needed, and will help inform the interpretation of isotopic data from diverse laboratory and field studies.

## Materials and Methods

We conducted a literature search using ISI Web of Science (search terms: carbon, nitrogen, sulfur, stable isotope, turnover, half-life) for studies that estimated, or contained data that could be used to estimate carbon, nitrogen and sulfur isotopic half-life (λ) for any animal taxa and tissue type. The 'references' sections of relevant studies were used to identify additional studies for inclusion.

Isotopic turnover rate can be estimated by modeling tissue stable isotope ratios as a function of either time or body mass using one of several basic modeling frameworks (S1 Text): Hobson and Clark [19], Hesslein et al. [20], and Fry and Arnold [10], all of which generally provide similar results, but vary in their appropriateness depending on the situation (i.e., growing versus non-growing animals). Several recent studies have evaluated whether multi-compartment models do a better job at describing isotopic turnover that single-compartment models [21,22]. Results have been equivocal. For these studies, we used the turnover estimate from the approach that was best able to describe the isotopic data from an information-theoretic approach (AIC).

**Fig 1:**
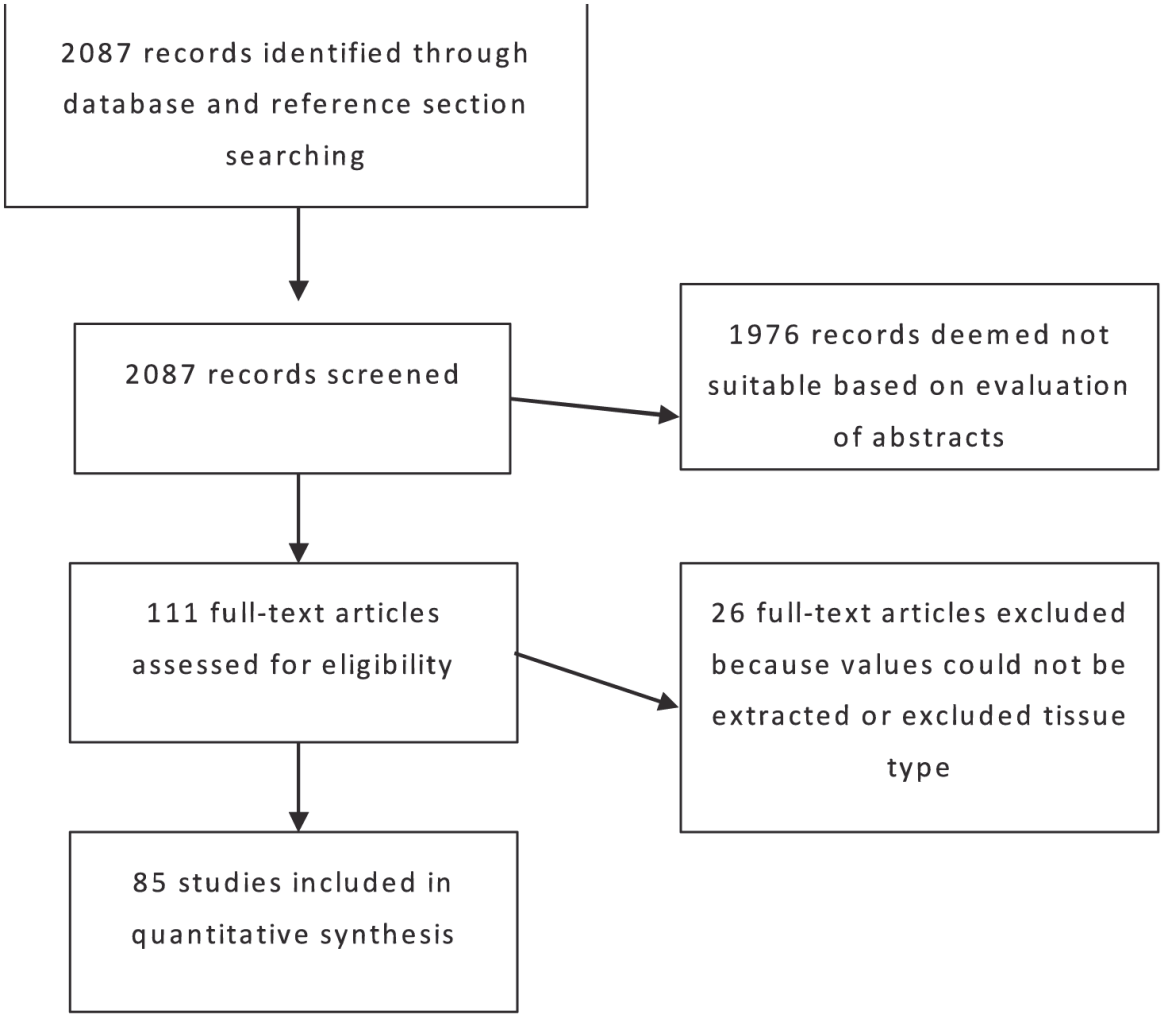
Flow diagram of manuscript screening and eligibility for this literature synthesis.

Animals included in this study span about 10 orders of magnitude in body mass (whiteleg shrimp, 0.009 mg to cows, 493 kg). We collected turnover estimates for a broad range of tissue types. Turnover estimates were arranged into five tissue groups: muscle, whole body, blood, blood plasma, and internal organs. The following tissue types either had small sample sizes or did not fit with our framework and were thus excluded from further analysis: gills, bone collagen, gonads, fins, eye, hair, scales, skin, and scute tissues. The tissue group ‘internal organs’ was quite broad and included the following tissue types: liver, brain, kidney, pancreas, spleen, lung, gastro-intestinal, and heart. Further evaluation of heterogeneity in isotopic turnover among organ types revealed no overall body size effect, and a high degree of overlap among the different internal organs. For the purpose of this analysis, we treated these different organs as a single group, though recognizing that individual internal organ types could differ with regard to isotopic turnover. Our final dataset included 486 isotopic turnover estimates from 86 species, taken from 85 separate peer-reviewed studies published from 1982–2014 (Fig. 1; S1 Table).

Where possible, we recalculated the reported half-life estimates to verify reported values, and calculated half-life where turnover or half-life values were not reported. Most estimates were derived from laboratory-based diet shift experiments or isotopic label uptake/depuration experiments. We also included a small number of field situations that very clearly mimicked an experimental diet shift [14,23,24]. Isotopic turnover λ was expressed as isotopic half-live, ln(2)/ λ, which is the time (in days) required for 50% equilibration with the experimental diet. We chose to present results as isotopic half-life because they are more readily interpretable and intuitive than isotopic turnover.

Temperature values are the reported average ambient temperature where the animal was housed during the diet shift study. We used animal mass at the start of the diet shift study as our indicator of body mass. While perhaps an imperfect measure, it was the only consistently reported measure of animal body mass in the original studies. For the few studies that did not report the starting body mass of the experimental animals, this value was estimated from the literature for the same or closely-related species at the same life-history stage. We excluded half-life estimates which lacked sufficient information to estimate body mass.

### Statistical approach

We hypothesized that isotopic half-life would increase as a function of animal body mass. In addition, we expected half-life and the relationship with body mass to potentially vary with factors such as taxon, tissue type, isotope, and temperature. Small sample sizes for certain taxa and tissue types, and a limited range of body sizes for some taxa, limited our ability to develop robust empirical models that are broadly applicable across all taxa and tissues. We developed models of half-life that used tissue type-taxon combinations as a categorical variable, and body mass as the covariate within an ANCOVA framework. We created a 5x4 contingency table (matrix) of our key categorical variables: tissue type (muscle, whole body, internal organs, blood, blood plasma) and taxon (invertebrates, vertebrate ectotherms, birds, mammals). Tissue type-taxon combinations (hereafter called ‘groups’) varied widely in sample size, with several tissue-taxon combinations represented by few or no half-life estimates. Groups with four or fewer half-life estimates were excluded from group-specific comparisons since it was deemed that this would be the absolute minimum sample size needed to reasonably estimate a slope and intercept within an ANCOVA framework. Body mass and half-life values were natural log transformed prior to analysis. Statistical analyses were conducted in SAS v 9.3 (Cary, NC, USA).

## Results

### Broad patterns

**Fig 2:**
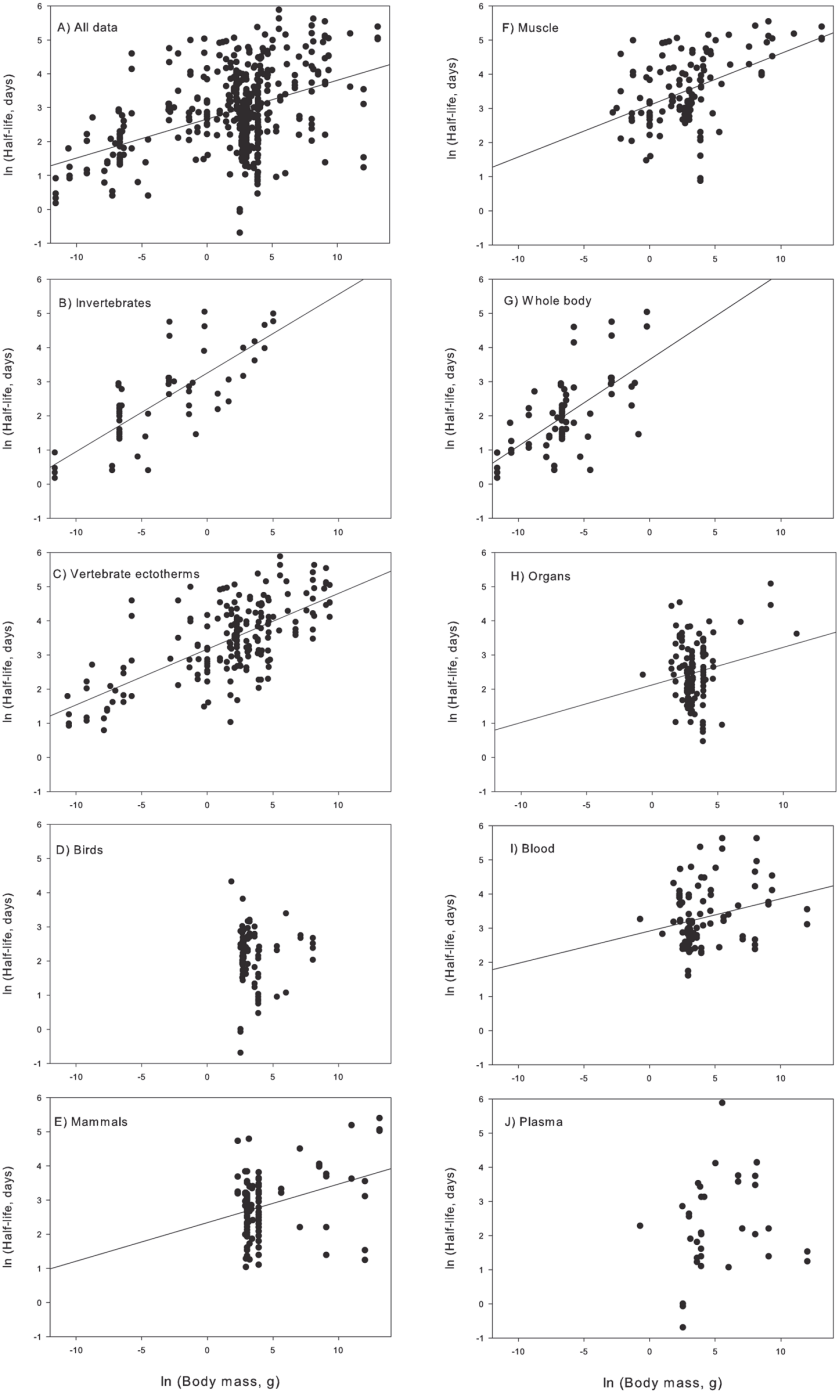
Relationships between ln(animal body mass, grams) and ln(isotopic half-life, days). A) Plot for all tissue types and taxonomic groups combined. B–E) Plotted separately for each taxon. F–J) Plotted separately for each tissue type. Regression information for each tissue type and taxa are presented in Table 1.

In the most inclusive analysis that includes half-life estimates for all isotopes (δ^13^C, δ^15^N, δ^34^S), taxa, and tissue types, half-life increased as a function of animal body mass (Fig. 2A):

ln(half-life) = 0.11 * ln(body mass) + 2.66, n = 486, p <0.0001, F = 117.02, RMSE = 1.03, r^2^ = 0.19 (Eq. 1).

Adding ‘isotope’ (δ^13^C, δ^15^N, δ^34^S) as a categorical variable to the above model revealed marginal significance (p = 0.06), and no significant body mass-isotope interaction (p = 0.29). Inclusion of ‘isotope’ did not notably reduce model error (RMSE = 1.02). As a result, half-life estimates for these three stable isotopes were hereafter combined.

We examined half-life as a function of body mass separately for each broadly-defined taxa (Fig. 2B–E) and tissue type (Fig. 2F–J), and present individual regression models for broad taxa and tissue types (Table 1). To examine more subtle patterns among broad taxa and tissue types, we separated half-life estimates into groups based on tissue-taxon combinations (hereafter referred to as ‘group’; Table 2).

**Table 1:** Regression coefficients of relationships between ln(animal body mass, g) and ln(isotopic half-life, days) for each taxon and tissue type included in this study.

	slope	intercept	F	p	N
Taxon					
Invertebrates	0.23	3.25	92.87	<0.0001	60
Vertebrate ectotherms	0.16	3.17	181.63	<0.0001	206
Birds	0.018	2.05	0.1	0.76	96
Mammals	0.11	2.33	15.76	<0.0001	124
**Tissue type**					
Muscle	0.16	3.11	45.75	<0.0001	144
Whole body	0.25	3.65	51.16	<0.0001	72
Organs	0.11	2.11	4.34	0.039	134
Blood	0.10	2.88	8.38	0.0047	100
Plasma	0.10	1.82	1.39	0.25	36

**Table 2:** The number of half-life estimates for each tissue type-taxon combination (hereafter referred to as ‘group’).

	Taxon				
Tissue type	Invertebrates	Vertebrate ectotherms^1^	Birds	Mammals	*total*
Muscle	11	88	16	29	*144*
Whole body	44	28	0	0	*72*
Organs	4*	37	43	50	*134*
Blood	1*	36	28	35	*100*
Plasma	0	17	9	10	*36*
*total*	*60*	*206*	*96*	*124*	***486***

Two groups (invertebrate organs and invertebrate blood; indicated by *) were excluded from group-level comparisons due to low sample size.

^1^fishes, reptiles, and amphibians

### Body mass–half-life relationships for ectotherms

For ectotherms, a model predicting half-life using body mass and tissue-taxon combination (group) revealed significant effects of body mass (p<0.0001) and ‘group’ (p<0.0001), but no significant interaction term (p = 0.28). Thus a model in which groups share a common allometric slope and group-specific intercepts (Table 3) describes the data: ln(half-life) = 0.22 * ln(body mass) + [group-specific intercept from Table 3]; n = 261, p <0.0001, F = 634.00, RMSE = 0.76, r^2^ = 0.63 (Eq. 2).

**Table 3:** Results of ANCOVA model for ectotherms.

Group	N	Intercept	Standard error
Vertebrate ectotherm plasma^1^	17	2.35	0.21
Vertebrate ectotherm organs^1^	37	2.48	0.14
Vertebrate ectotherm blood^2^	36	3.08	0.15
Invertebrate muscle^2^	11	3.13	0.23
Vertebrate ectotherm muscle^2^	88	3.28	0.09
Invertebrate whole body^2^	44	3.28	0.16
Vertebrate ectotherm whole body^3^	28	3.65	0.20

Half-life for ectotherm groups (tissue type-taxon combinations) was described by a model with a common slope (0.22) with body mass and group-specific intercepts.

^1–3^indicates groupings based on the absence of significant differences (p = 0.05) among group-specific intercepts.

Pairwise comparison of intercepts and associated standard errors revealed that many group intercepts were not statistically different from each other, producing three significantly different and distinct clusters (indicated by superscript numbers 1–3 in Table 3). Vertebrate ectotherm plasma and organs had the lowest intercepts (i.e., lowest half-life), and were not significantly different from each other (‘1’ in Table 3). The second grouping was comprised of vertebrate ectotherm blood and muscle, and invertebrate muscle and whole body (‘2’ in Table 3). Vertebrate ectotherm whole body was the final grouping (‘3’ in Table 3).

**Fig 3:**
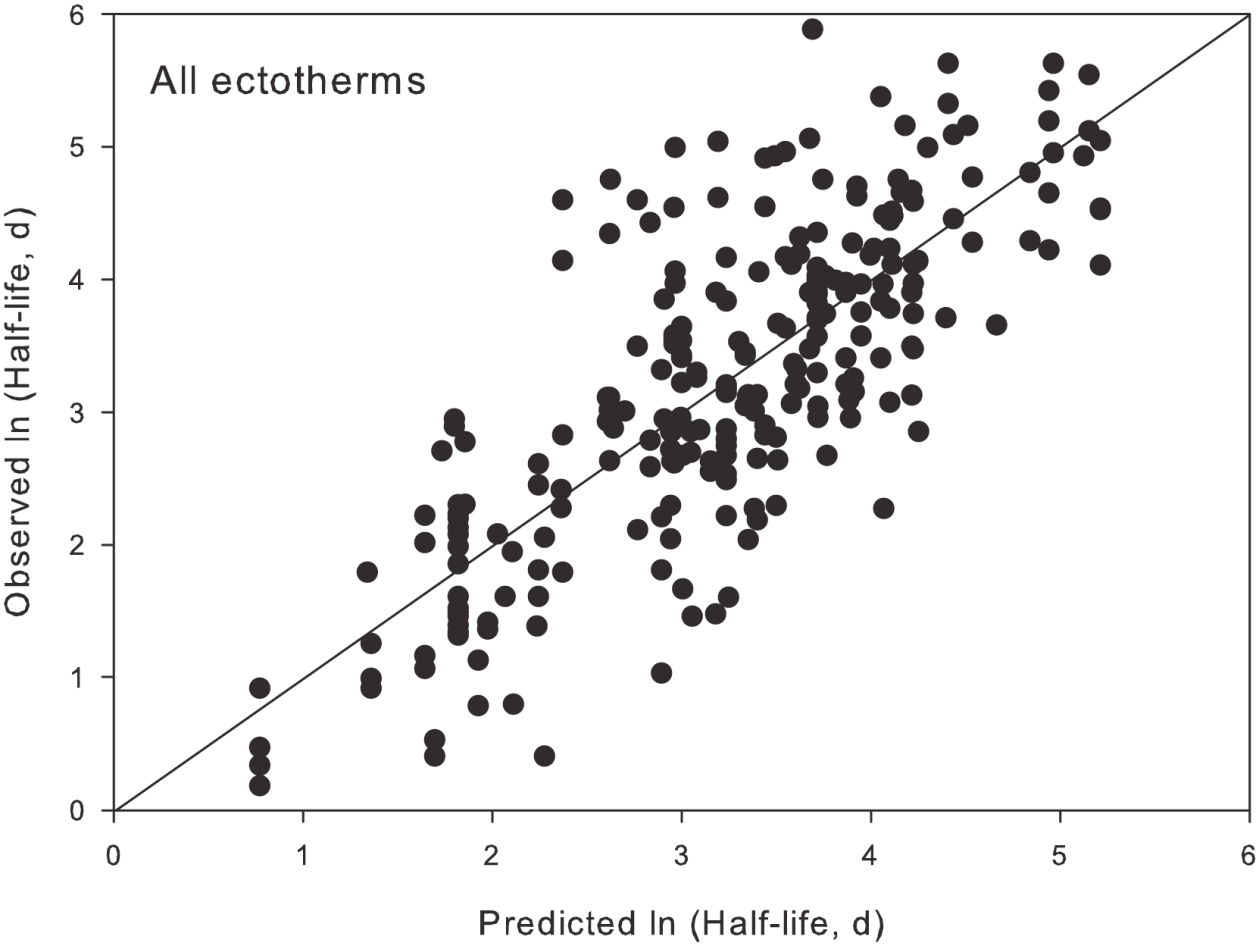
Predicted versus observed ln(half-life) for ectotherms from our model with a common body mass slope and group-specific intercepts (Eq. 3).

As an alternative to using the individual group-specific intercepts (Eq. 2), we combined groups that were not significantly different from each other for a simplified model of half-life for ectotherms: ln (half-life) = 0.21*ln (body mass) + [2.47 (vertebrate ectotherm plasma & organs), 3.23 (vertebrate ectotherm blood and muscle & invertebrate muscle and whole body), 3.60 (vertebrate ectotherm whole body)]; n = 261, p<0.0001, F = 1276.3, RMSE = 0.76, r^2^ = 0.63 (Eq. 3). A comparison of observed and predicted half-life values for ectotherms (from Eq. 3) shows that predicted values fall on the 1:1 line (Fig. 3).

Finally, considering that ectotherm metabolism and a wide range of other rates are strongly temperature dependent [18], we tested whether temperature explained any additional variance in the body mass-half-life relationship for ectotherms. The effect of temperature was weak but statistically significant (p = 0.02), and had a small effect of the model RMSE (reduced from 0.76 to 0.73). Overall our results indicate a relatively minor effect of temperature on isotopic half-life for ectotherms.

### Body mass–half-life relationships for endotherms

For endotherms, there was a weak positive relationship between half-life and body mass: ln(half-life) = 0.13 * ln(body mass) + 2.01; n = 220, p <0.0001, F = 24.15, RMSE = 0.87, r^2^ = 0.10 (4)

Comparison among groups revealed heterogeneous slopes and intercepts—a general model including body mass and tissue-taxon combination (group) revealed significant effects of ‘group’ (p<0.0001), and a significant body mass*group interaction term (p<0.0001). The significant interaction term thus precludes use of a general model with a common slope.

**Fig 4:**
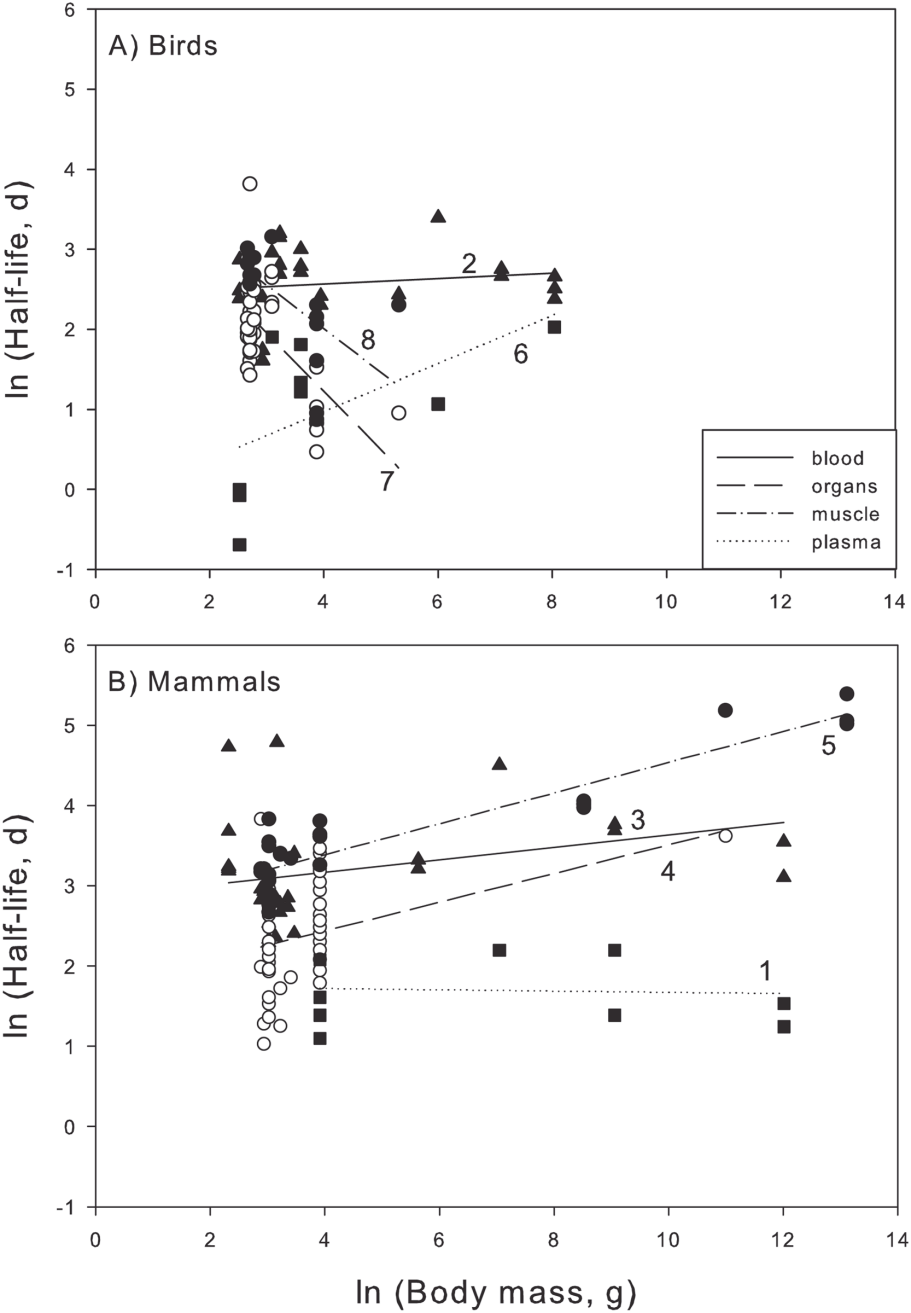
Relationships between ln(body mass) and ln(half-life) for tissue types in A) birds and B) mammals. Numbers correspond with Table 4. Symbols: filled triangles = blood; open circles = organs, filled circles = muscle, filled squares = plasma.

For three groups: mammal plasma, mammal blood, and bird blood there was no significant relationship with body mass (labeled 1,2, and 3 in Table 4 and Fig. 4). Thus half-life for these groups can simply be approximated as the average (ln(half-life): mammal plasma = 1.69, mammal blood = 3.19, bird blood = 2.57). For mammal organs, mammal muscle, and bird plasma (4,5, and 6), half-life increased significantly with body mass, and can be estimated from group-specific equations (Table 4 and Fig. 4). Model slopes and intercepts for these three groups differed notably. Finally, for bird organs and bird muscle, there was a negative relationship between half-life and body mass (7 and 8 in Table 4 and Fig. 4). Both of these groups had an exceedingly narrow range in body mass (Fig. 4). As a result we have less confidence in the slopes and intercepts reported in Table 4.

**Table 4:** Group (tissue-taxa combination) intercepts and slopes (including standard errors) for body mass—half-life relationships for endothermic groups.

Group	Slope	Standard error	Intercept	Standard error	Estimated half-life for a 50 gram animal (days)
*Non-significant slope*					
1) Mammal plasma	**-0.01**	0.06	1.75	0.43	5.6
2) Bird blood	**0.03**	0.08	2.43	0.25	13.1
3) Mammal blood	**0.08**	0.07	2.86	0.18	23.9
*Positive slope*					
4) Mammal organs	**0.18** *	0.09	1.72	0.26	11.3
5) Mammal muscle	**0.19** **	0.06	2.62	0.18	29.4
6) Bird plasma	**0.30** **	0.12	-0.23	0.44	2.6
*Negative slope*					
7) Bird organs	**-0.73** ***	0.16	4.13	0.46	3.6
8) Bird muscle	**-0.55** **	0.19	4.19	0.63	7.9

To facilitate an overall comparison among tissue-taxa combinations, the group-specific equation was used to estimate half-life (days) for a standardized 50 g animal (ln(50) = 3.91).

*Significantly different from 0 at the p<0.05 level

** Significant at the p<0.01 level

*** Significant at the p<0.0001 level

To facilitate a more general comparison of endothermic groups, we estimated half-life for a hypothetical 50 gram animal for each group (Table 4, last column). For mammals, half-life was highest for muscle, followed by blood, organs, and plasma. For birds, blood had the highest half-life, followed by muscle, organs, and blood plasma. Overall, half-lives for mammal tissues ranged from 1.8 to 3.7x higher than for birds.

## Discussion

It is widely recognized that stable isotopes can provide a time-integrated depiction of consumer diet and trophic relationships, and that the time-integrative nature of the approach is a valuable attribute of the stable isotope approach [2,25]. Perhaps so, though a largely unresolved question is ‘over what time period is resource use integrated’? Many food webs are highly size-structured. For example, in aquatic food webs animal body mass increases many orders of magnitude from the base of the food web to top predators. Since the time period of isotopic integration scales allometrically with body mass (as we demonstrate here), the result is simultaneous isotopic integration at multiple temporal scales within a single food web. Using the pelagic food chain of Lake Tanganyika as an example, O’Reilly et al. [9] discussed how failure to consider that animal tissues from different trophic levels integrate at different time scales can confound the ecological interpretation of stable isotope data. Considering that many stable isotope field studies, such as those using stable isotope mixing models [8] tacitly assume isotopic steady state between resources and consumers, we believe the issue of isotopic time averaging is inadequately considered in many field studies that use stable isotopes. This general concern motivated our effort to synthesize the published isotopic half-life estimates in an attempt to make broad-scale statistical generalities about how half-life varies with factors such as body mass, taxa, and tissue type.

### Determinants of half-life

The rate of isotopic incorporation for a given tissue is a consequence of two distinct processes: accrual of new biomass (growth) and catabolic tissue replacement (catabolic turnover) [10,20]. For tissue growth, the isotopic value of new tissue gradually dilutes that of the existing biomass pool. Catabolic turnover involves elemental turnover within a tissue independent of tissue growth, and has been proposed to correspond somewhat closely with the rate of protein turnover [15,17]. Growth is expected to dominate turnover in small, rapidly growing animals, while catabolic turnover would likely dominate for slow growing animals, and the relative importance of catabolic turnover is expected to increase with animal body size [26]. Of course, body mass and growth rate can also be uncoupled. For example, consider two same-sized animals of different species. The first animal has reached its maximum body size, while the second animal is a rapidly growing juvenile. One would expect the second to have a notably higher isotopic turnover rate than the first due to the contributions of both growth and catabolic processes to isotopic turnover for the second animal. This hypothetical example highlights the importance of considering the underlying processes responsible for isotopic turnover, and may help explain the relatively high degree of variation in half-life at a given body mass. While some lab experiments do partition turnover into growth and catabolic components, most do not. Though it would perhaps be informative to examine, we did not further evaluate the relative importance of these contributing processes, but instead focused on examining broad patterns of isotopic half-life.

Our modeling approach employed body mass as a covariate, and combinations of tissue type and taxa as a categorical variable. It is well-known that different tissues from the same organism can have distinct rates of isotopic incorporation [27,28], presumably indicative of differential rates of protein turnover [1,15]. Furthermore, we would expect differences in turnover among taxonomic groups (invertebrates, fishes, mammals, birds), largely reflective of more rapid turnover rates for endothermic animals due to their higher feeding and metabolic rates (metabolism broadly defined). We examined the roles of tissue type and taxon by considering combinations of tissue categories (muscle, whole body, organs, blood, blood plasma) and taxon (invertebrates, vertebrate ectotherms, birds, mammals; Table 2). Several tissue-taxon combinations had either few or no observations, or else little variation in body mass. Sufficient data were available to characterize 13 of the 20 possible tissue-taxon combinations.

We used an allometric approach to examine variability in half-lives of animal tissues. Intuitively, we would expect half-life to increase with body mass: a small animal will likely have high specific growth rates and possibly high specific protein turnover rates, such that an isotopic measurement would reflect several days or weeks of feeding. In contrast, a large animal would be expected to have a low specific growth rate and protein turnover rate, such that an isotopic measurement would be expected to integrate over a period of months to years. More formally, Carleton and Martinez del Rio (2005) hypothesized that turnover λ can be interpreted as v/P, where v is the net rate of influx of an element into a tissue, and P is the size of the element pool. v is expected to be proportional to body mass^3/4^, while P should scale isometrically with body mass, such that turnover should be proportional to body mass^-1/4^ (i.e., mass^3/4^/mass). In addition, specific growth rates also tend to scale with body mass^-1/4^ [29]. Thus, both processes that contribute to isotopic turnover (tissue growth and catabolic turnover) are expected to scale with body mass with an exponent of-1/4.

### Ectotherms

Half-life for ectotherms was described by a model with a common slope and group-specific intercepts. The body mass-half-life relationship had a slope of ~ 0.22 (equivalent to a turnover exponent of-0.22), which corresponds reasonably well with the predicted slope of ~0.25 [1,15], as well as with the observed allometric slope for carbon isotopes in fish muscle [14].

We estimated group-specific intercepts within an ANCOVA framework (Eq. 2; Table 3), and subsequently simplified the model by combining groups that were not significantly different from each other (Eq. 3; Table 3, superscript numbers). Vertebrate ectotherm organs and blood plasma had the lowest half-lives. This finding corresponds with numerous other studies reporting that fish internal organs and blood plasma turn over rapidly compared to fish blood or muscle tissue [28,30,31].The remaining ectothermic groups (with the exception of vertebrate ectotherm whole body) had statistically indistinguishable intercepts, thereby indicating a common allometric relationship. Vertebrate ectotherm whole body had a marginally significant higher intercept than the other groups (Table 3). Studies generally use whole body measurements for animals that are too small to separate individual tissues. Thus there are only ‘whole body’ estimates for invertebrates and very small fish, and there is little overlap in body mass between whole body and other estimates (compare range in body mass in Fig. 2G with that of other tissue types). We opted to keep vertebrate ectotherm whole body estimates separate from the other groups in Eq. 3, but we note that this difference could simply be a result of limited overlap in body mass.

### Endotherms

In contrast with ectotherms, endotherm tissue-taxa combinations exhibited group-specific slopes and intercepts (Table 4; Fig. 4). Thus, while we can still provide a statistical model for approximating half-life (Table 4), interpretation is less straightforward than for ectotherms.

For three groups (mammal plasma, mammal blood, and bird blood), there was no significant change in half-life with animal body mass (Table 4; Fig. 4). This result runs counter to our expectation of a positive relationship between turnover and body mass, and suggests that half-life for these groups can be evaluated without reference to body mass. In contrast with our findings, two earlier studies reported a positive relationship between half-life and body mass for bird blood [15,16]. Reasons for these differences are unclear, though we note that all inferences are based on relatively small sample sizes. No previous studies have examined whether isotopic half-life is related to body mass for mammal tissues of any type.

For three endotherm groups (mammal organs, mammal muscle, and bird plasma) there was the expected positive relationship between body mass and half-life, with slopes ranging from 0.18 to 0.3. Allometric slopes for mammal muscle and mammal organs were similar, though mammal muscle had a higher intercept (Table 4, Fig. 4B). This finding is consistent with previous literature indicating that internal organs tend to have more rapid turnover than muscle tissue (Tieszen et al. 1983). While the allometric slope for bird plasma was high (0.3), bird plasma half-life was low compared to other tissues (Table 4, Fig. 4A). It is puzzling that mammal plasma and bird plasma had such different body mass slopes (-0.01 versus 0.30). Nevertheless, our results indicate that blood plasma has a short half-life compared to blood generally, which was expected since blood plasma cells are short-lived compared to red and white blood cells.

Finally, for bird organs and bird muscle, there was actually a negative relationship between body mass and half-life (Table 4; Fig. 4A). This result was unexpected. For these two groups, there was little variation in body mass (Fig. 4), and it possible that our results are instead driven by small samples size and one or a small number of anomalous data points. Allometric relationships for bird muscle and bird organs clearly warrant further investigation.

### Isotope-specific differences

We found marginal differences in the body mass-half-life relationship among the three isotopes we evaluated (δ^13^C, δ^15^N, and δ^34^S). This result is somewhat surprising, considering the unique metabolic pathways, biochemical roles and functions of these three elements. Individual studies have found differences in half-life between carbon and nitrogen isotopes, though the direction of these trends has not been consistent. Carleton and Martinez del Rio [15] found nitrogen half-life for birds to be 50% higher than for carbon. In contrast, a study of juvenile steelhead found nitrogen half-life to be lower than carbon [32]. Other studies report no difference between half-life for δ^13^C and δ^15^N [33]. Compared to carbon and nitrogen, sulfur occurs at low concentrations in animal tissues, and most sulfur is bound within proteins (amino acids cysteine and methionine). We note that there were relatively few half-life estimates for δ^34^S (18 half-life estimates from six separate papers, versus 468 combined estimates for carbon and nitrogen), and most sulfur estimates were from a single study comparing δ^13^C, δ^15^N and δ^34^S turnover for a suite of mice tissues (Arneson et al. 2006). Individual studies involving sulfur isotopes have found that half-life estimates for sulfur tend not to differ notably from other elements (Tarboush 2006, Arneson et al. 2006, Bahar 2009, Hesslein et al. 1993, MacAvoy 2001). Despite the profound biochemical differences among these three elements, there were no striking differences in allometric relationships among them. Overall, differences among isotopes appear to be a minor source of noise within the broader context of a rather general allometric relationship.

### Temperature effect

Though biological rates tend to be strongly influenced by temperature [18], there was a weak (p = 0.02) effect of temperature on half-life for ectotherms, after taking into account body mass. Our sample size including temperature data was quite large (247 estimates), and the range in temperature was large (approximately 35°C). Similarly, Weidel et al. [14] found virtually no effect of temperature on carbon isotope half-life in fish muscle tissue. In contrast, individual experiments that included temperature as a treatment have sometimes found an effect of temperature on half-life. For example Bosley et al. [34] and Witting et al. [35] found that rearing larval fish at higher water temperatures resulted in lower carbon half-life. This may have been due to a positive effect of temperature on growth rate, which is expected to dominate isotopic turnover in rapidly growing animals. Overall, our results indicate that the effect of temperature on half-life is small within the context of the overall variation in half-life.

### Model predictions and error

Our empirical models predicting isotopic half-life for animal tissues are based on a large body of experimental data from animals spanning a range of body sizes. Our model can be used as a tool to directly approximate the time scale of diet integration from body size, tissue type, and taxonomic group. As an example, using Eq. 3, the half-life of muscle tissue from a 10 gram vertebrate ectotherm (group 2 in Table 3) is estimated to be 44 days, with a 95% prediction interval ranging from 10 to 196 days. As another example, muscle tissue from a 10 gram mammal is estimated at 21 days (95% prediction interval is 12–38 days). While our model allows prediction of half-life from body mass and tissue type, the wide prediction intervals highlight the fact that this approach provides a coarse approximation of isotopic half-life.

The high degree of model uncertainty stems directly from the high degree of variability among the original half-life estimates—in other words, animals of the same size, tissue type, and broad taxonomic group can have widely divergent isotopic half-lives. The diet shift experiments included in our synthesis were conducted by a wide range of investigators, and under a great range of experimental conditions (diets, growth rates, temperatures, etc.). This itself may help explain the high degree of noise in the body mass—half-life relationship. The general implication is that a half-life estimate from a single experimental study cannot be used as a basis for accurately inferring half-life in a field situation. Our approach of synthesizing the many published half-life estimates (literature review current to May 2014) allows us to make more rigorous statistical generalities, and to explicitly recognize the high degree of variability, which in this case translates to model uncertainty.

A number of factors not explicitly considered in our study may affect the dynamics of isotopic incorporation. These include differences in animal growth rates, diet and food quality, life history stage, physiological state, and isotopic routing [1,36,37]. In addition, the original estimates of half-life themselves are subject to several sources of error. These could include insufficient isotopic difference between experimental diets, error in the assumed diet-tissue enrichment factor, failure to run the experiment to isotopic steady-state, and use of a fundamentally inappropriate model form. As an example of the latter, several models used to estimate half-life assume exponential somatic growth of the animal in the diet switch experiment–deviation from this assumption will result in erroneous half-life estimates. In effect, there is uncertainty associated with half-life value reported in the original studies, though this uncertainty was rarely estimated or reported, and is not explicitly considered here.

Other variables used in our analysis were also subject to error. For example, we used animal body mass at the start of the experiment as the measure of body mass in our analysis. While this is an imperfect measure of animal body mass, it was the best and most reliable option given the available data.

### Summary

Our goal was to evaluate whether broad empirical generalities can be made from the large numbers of half-life estimates that have been published over the past few decades. To provide a basis for comparison, there have been several broad empirical syntheses of diet-tissue discrimination factors [4–6]. These syntheses subsequently provided the foundation for the quantitative application of stable isotope approaches, which typically require making explicit assumptions about diet-tissue discrimination. Diet-tissue discrimination and its variability are now reasonably well-characterized, and studies routinely incorporate the observed variability in diet-tissue discrimination into mixing model outputs. In contrast, the topic of isotopic turnover and half-life has not been the subject of broad empirical syntheses. Consideration of the time scale of isotopic incorporation in animal tissues can be vitally important in the interpretation of stable isotope results [9,36]. For ectotherms, half-life increased predictably with body size, producing a relatively simple empirical model for approximating half-life. For birds and mammals, the relationship with body mass involved different allometric slopes and intercepts for different tissues. Despite there being some broad empirical patterns, there was also substantial unexplained variation in our models of isotopic half-life, indicating that measurement error and unmeasured factors affect isotopic half-life. Our hope was that a broad-scale synthesis of the existing data could reveal general patterns that could not have been detected from individual studies. While our comparative approach cannot directly elucidate underlying mechanisms, it does reveal gaps in our knowledge and understanding that will hopefully inform future experimental work at this essential interface of ecology and physiology.

## Supporting Information

S1 Text Background information on the modeling approaches used to estimate isotopic half-life. (DOCX)

S1 Table Data sources and associated information for the half-life estimates included in this study. (DOCX)
