# Supplementary material for: Stable Isotope Turnover and Half-Life in Animal Tissues: A Literature Synthesis
Source: PLoS One. 2015 Jan 30;10(1):e0116182. doi: 10.1371/journal.pone.0116182 (PMC4321325; doi:10.1371/journal.pone.0116182)
Supplement: S1 Text — (DOCX) [file pone.0116182.s001.docx]

**Text S1**

Nearly all published estimates of isotope turnover or half-life used a one-compartment modeling approach to estimate isotopic turnover and half-life in animal tissues. While there has been recent interest in two compartment models [[1](#_ENREF_1),[2](#_ENREF_2)], this more complex approach is not easily applicable to much of the literature data that are available. Isotopic turnover is typically estimated by modeling tissue stable isotope ratios as a function of either time or body mass using one of three models, as described in Hobson and Clark [[3](#_ENREF_3)], Hesslein et al. [[4](#_ENREF_4)], and Fry and Arnold [[5](#_ENREF_5)]. Here we briefly review each of the three model structures used for estimating half-life. In all three models, newly accreted tissue following the diet shift and catabolic tissue turnover of existing body tissues (independent of somatic growth) shift the animal towards equilibrium with the new diet. These three models differ only in how isotopic change is expressed, and whether isotopic change is explicitly partitioned into growth and metabolic turnover components.

The Hobson and Clark model expresses isotopic turnover as an exponential function of time, such that the turnover parameter (λ; see parameter descriptions in Table 1) combines the growth and metabolic tissue turnover:

[1] δ_t_ = δ_n_ + (δ_o_-δ_n_)e^-(λ)t^

The model used by Hesslein et al. [[4](#_ENREF_4)] is similar, except λ is partitioned into growth (*k*) and metabolic tissue turnover (*m*). Provided the growth rate (*k*) is known, the parameter *m* can be fit using least squares:

[2] δ_t_ = δ_n_ + (δ_o_-δ_n_)e^-(k+m)t^

For both of the above models, isotopic half-life (days), defined as the time required for a tissue to reach 50% equilibration with the isotopic value of a new diet, can be estimated as ln(2)/λ. Finally, the Fry and Arnold model fits a power function to estimate a turnover parameter, *c*:

[3] δ_m_ = δ_n_ + (δ_o_-δ_n_)*w^c^

A turnover function T(w) expressed in terms of mass is estimated as T(w) = w^(c+1)^, where w is the percent of initial body mass at the time of sampling (w_n_/w_o_*100). Half-life is estimated by setting T(w) = 0.5, and solving for w. This can be expressed in terms of time if growth rate is known.

Table 1. Parameters from the three basic models used to estimate isotopic turnover and half-life. Symbols and variable names differ slightly from the original studies.

| *symbol* | *Definition* |
| --- | --- |
| *δ_t_* | isotopic value (‰) of the organism at time t |
| *δ_o_* | initial isotopic value (‰) at equilibrium with the old diet |
| *δ_n_* | isotopic value (‰) after equilibration with the new diet |
| *t* | time (days) |
| *λ* | Turnover rate (days^-1^) |
| *k* | Growth rate (days^-1^) |
| *m* | Metabolic turnover (days^-1^) |
| *δ_w_* | Isotopic value (‰) of the organism at mass w |
| *w_o_* | initial mass (g) |
| *w_n_* | mass at the time of sampling (g) |
| *w* | percent of initial body mass at the time of sampling |

**References**

1. Carleton SA, Kelly L, Anderson-Sprecher R, del Rio CM (2008) Should we use one-, or multi-compartment models to describe C-13 incorporation into animal tissues? Rapid Communications in Mass Spectrometry 22: 3008-3014.

2. Cerling TE, Ayliffe LK, Dearing MD, Ehleringer JR, Passey BH, et al. (2007) Determining biological tissue turnover using stable isotopes: the reaction progress variable. Oecologia 151: 175-189.

3. Hobson KA, Clark RG (1992) Assessing avian diets using stable isotopes I: Turnover of 13C in tissues. Condor 94: 181-188.

4. Hesslein RH, Hallard KA, Ramlal P (1993) Replacement of sulfur, carbon, and nitrogen in tissue of growing broad whitefish (*Coregonus nasus*) in response to a change in diet traced by 34S, 13C, and 15N. Canadian Journal of Fisheries and Aquatic Sciences 50: 2071-2076.

5. Fry B, Arnold C (1982) Rapid 13C/12C turnover during growth of brown shrimp (*Penaeus aztecus*). Oecologia 54: 200-204.
