# Supplementary material for: Stable Isotope Turnover and Half-Life in Animal Tissues: A Literature Synthesis
Source: PLoS One. 2015 Jan 30;10(1):e0116182. doi: 10.1371/journal.pone.0116182 (PMC4321325; doi:10.1371/journal.pone.0116182)
Supplement: S1 Table — (DOCX) [file pone.0116182.s002.docx]

Table S1. Data sources and associated information for the published half-life estimates included in this study.

| \| **Common name** \| **Scientific name** \| **Tissue** \| **Isotope** \| **Start weight (g)** \| **Temp (°C)** \| **Half-life (d)** \| **Reference** \| \| --- \| --- \| --- \| --- \| --- \| --- \| --- \| --- \| \| African Penguin \| *Spheniscus demersus* \| blood \| 15N \| 3100 \|  \| 10.8 \| Barquete et al. 2013 \| \| African Penguin \| *Spheniscus demersus* \| blood \| 15N \| 3100 \|  \| 12.3 \| Barquete et al. 2013 \| \| African Penguin \| *Spheniscus demersus* \| red blood cells \| 15N \| 3100 \|  \| 14.3 \| Barquete et al. 2013 \| \| African Penguin \| *Spheniscus demersus* \| plasma \| 15N \| 3100 \|  \| 7.6 \| Barquete et al. 2013 \| \| Alpaca \| *Lama pacos* \| liver \| 13C \| 59400 \|  \| 37.3 \| Sponheimer et al. 2006 \| \| Alpaca \| *Lama pacos* \| muscle \| 13C \| 59400 \|  \| 178.7 \| Sponheimer et al. 2006 \| \| Amazon River Frog \| *Rana palmipes* \| muscle \| 15N \| 3.36 \| 23.5 \| 138.6 \| McIntyre & Flecker 2006 \| \| American Alligator \| *Alligator mississippiensis* \| red blood cells \| 13C \| 3500 \|  \| 141.5 \| Rosenblatt & Heithaus 2012 \| \| American Alligator \| *Alligator mississippiensis* \| red blood cells \| 15N \| 3500 \|  \| 227.3 \| Rosenblatt & Heithaus 2012 \| \| American Alligator \| *Alligator mississippiensis* \| plasma \| 13C \| 3500 \|  \| 63.0 \| Rosenblatt & Heithaus 2012 \| \| American Alligator \| *Alligator mississippiensis* \| plasma \| 15N \| 3500 \|  \| 62.4 \| Rosenblatt & Heithaus 2012 \| \| American Crow \| *Corvus brachyrhynchos* \| blood cells \| 13C \| 404 \|  \| 29.8 \| Hobson & Clark 1993 \| \| American Crow \| *Corvus brachyrhynchos* \| plasma \| 13C \| 404 \|  \| 2.9 \| Hobson & Clark 1993 \| \| Arctic Fox \| *Vulpes lagopus* \| blood cells \| 13C \| 8600 \|  \| 43.0 \| Lecomte et al. 2011 \| \| Arctic Fox \| *Vulpes lagopus* \| blood cells \| 15N \| 8600 \|  \| 40.0 \| Lecomte et al. 2011 \| \| Arctic Fox \| *Vulpes lagopus* \| plasma \| 13C \| 8600 \|  \| 9.0 \| Lecomte et al. 2011 \| \| Arctic Fox \| *Vulpes lagopus* \| plasma \| 15N \| 8600 \|  \| 4.0 \| Lecomte et al. 2011 \| \| Armored Catfish \| *Ancistrus triradiatus* \| blood \| 15N \| 2.64 \| 23.5 \| 16.9 \| McIntyre & Flecker 2006 \| \| Armored Catfish \| *Ancistrus triradiatus* \| muscle \| 15N \| 2.64 \| 23.5 \| 18.2 \| McIntyre & Flecker 2006 \| \| Armored Catfish \| *Pterygoplichthys disjunctivus* \| red blood cells \| 13C \| 1.71 \| 25 \| 88.57 \| German & Miles 2010 \| \| Armored Catfish \| *Pterygoplichthys disjunctivus* \| red blood cells \| 15N \| 1.71 \| 25 \| 9.69 \| German & Miles 2010 \| \| Armored Catfish \| *Pterygoplichthys disjunctivus* \| plasma \| 13C \| 1.71 \| 25 \| 22.84 \| German & Miles 2010 \| \| Armored Catfish \| *Pterygoplichthys disjunctivus* \| plasma \| 15N \| 1.71 \| 25 \| 7.66 \| German & Miles 2010 \| \| Atlantic Cod \| *Gadus morhua* \| blood \| 13C \| 9.6 \| 11.3 \| 54.2 \| Ankjaerø et al. 2012 \| \| Atlantic Cod \| *Gadus morhua* \| blood \| 13C \| 9.6 \| 11.3 \| 59.8 \| Ankjaerø et al. 2012 \| \| Atlantic Cod \| *Gadus morhua* \| blood \| 13C \| 9.6 \| 11.3 \| 48.9 \| Ankjaerø et al. 2012 \| \| Atlantic Cod \| *Gadus morhua* \| blood \| 15N \| 9.6 \| 11.3 \| 52.4 \| Ankjaerø et al. 2012 \| \| Atlantic Cod \| *Gadus morhua* \| blood \| 15N \| 9.6 \| 11.3 \| 49.7 \| Ankjaerø et al. 2012 \| \| Atlantic Cod \| *Gadus morhua* \| blood \| 15N \| 9.6 \| 11.3 \| 40.8 \| Ankjaerø et al. 2012 \| \| Atlantic Cod \| *Gadus morhua* \| heart \| 13C \| 9.6 \| 11.3 \| 30.2 \| Ankjaerø et al. 2012 \| \| Atlantic Cod \| *Gadus morhua* \| heart \| 13C \| 9.6 \| 11.3 \| 25.0 \| Ankjaerø et al. 2012 \| \| Atlantic Cod \| *Gadus morhua* \| heart \| 13C \| 9.6 \| 11.3 \| 38.2 \| Ankjaerø et al. 2012 \| \| Atlantic Cod \| *Gadus morhua* \| heart \| 15N \| 9.6 \| 11.3 \| 30.8 \| Ankjaerø et al. 2012 \| \| Atlantic Cod \| *Gadus morhua* \| heart \| 15N \| 9.6 \| 11.3 \| 30.3 \| Ankjaerø et al. 2012 \| \| Atlantic Cod \| *Gadus morhua* \| heart \| 15N \| 9.6 \| 11.3 \| 34.4 \| Ankjaerø et al. 2012 \| \| Atlantic Cod \| *Gadus morhua* \| muscle \| 13C \| 9.6 \| 11.3 \| 40.1 \| Ankjaerø et al. 2012 \| \| Atlantic Cod \| *Gadus morhua* \| muscle \| 13C \| 9.6 \| 11.3 \| 45.7 \| Ankjaerø et al. 2012 \| \| Atlantic Cod \| *Gadus morhua* \| muscle \| 13C \| 9.6 \| 11.3 \| 26.9 \| Ankjaerø et al. 2012 \| \| Atlantic Cod \| *Gadus morhua* \| muscle \| 15N \| 9.6 \| 11.3 \| 77.5 \| Ankjaerø et al. 2012 \| \| Atlantic Cod \| *Gadus morhua* \| muscle \| 15N \| 9.6 \| 11.3 \| 56.0 \| Ankjaerø et al. 2012 \| \| Atlantic Cod \| *Gadus morhua* \| muscle \| 15N \| 9.6 \| 11.3 \| 35.4 \| Ankjaerø et al. 2012 \| \| Atlantic Salmon \| *Salmo salar* \| muscle \| 13C \| 23.3 \|  \| 71.7 \| Jardine et al. 2004 \| \| Atlantic Salmon \| *Salmo salar* \| muscle \| 13C \| 21.4 \|  \| 21.9 \| Jardine et al. 2004 \| \| Atlantic Salmon \| *Salmo salar* \| muscle \| 13C \| 22.3 \|  \| 19.2 \| Jardine et al. 2004 \| \| Black Bear \| *Ursus americanus* \| red blood cells \| 13C \| 164400 \|  \| 34.7 \| Hilderbrand et al. 1996 \| \| Black Bear \| *Ursus americanus* \| red blood cells \| 15N \| 164400 \|  \| 22.4 \| Hilderbrand et al. 1996 \| \| Black Bear \| *Ursus americanus* \| blood plasma \| 13C \| 164400 \|  \| 4.6 \| Hilderbrand et al. 1996 \| \| Black Bear \| *Ursus americanus* \| blood plasma \| 15N \| 164400 \|  \| 3.5 \| Hilderbrand et al. 1996 \| \| Blackfly \| *Simulium vittatum* \| whole organism \| 15N \| 0.0007 \| 20 \| 1.5 \| Overmyer et al. 2008 \| \| Blackfly \| *Simulium vittatum* \| whole organism \| 15N \| 0.0007 \| 20 \| 1.7 \| Overmyer et al. 2008 \| \| Blue Cod \| *Parapercis colias* \| blood \| 15N \| 252.2 \| 12 \| 278.0 \| Suring & Wing 2009 \| \| Blue Cod \| *Parapercis colias* \| blood cells \| 15N \| 252.2 \| 12 \| 205.0 \| Suring & Wing 2009 \| \| Blue Cod \| *Parapercis colias* \| blood plasma \| 15N \| 252.2 \| 12 \| 359.0 \| Suring & Wing 2009 \| \| Blue Mussel \| *Mytilus edulis* \| muscle \| 13C \| 2.17 \| 15.9 \| 8.9 \| Dubois et al. 2007 \| \| Blue Mussel \| *Mytilus edulis* \| muscle \| 15N \| 2.17 \| 15.9 \| 14.1 \| Dubois et al. 2007 \| \| Bluegill \| *Lepomis macrochirus* \| muscle \| 13C \| 0.06 \| 21.5 \| 17.8 \| Weidel et al. 2011 \| \| Bluegill \| *Lepomis macrochirus* \| muscle \| 13C \| 5.38 \| 21.5 \| 28.9 \| Weidel et al. 2011 \| \| Bluegill \| *Lepomis macrochirus* \| muscle \| 13C \| 72.7 \| 21.5 \| 115.5 \| Weidel et al. 2011 \| \| Broad Whitefish \| *Coregonus nasus* \| muscle \| 13C \| 26 \| 10 \| 101.9 \| Hesslein et al. 1993 \| \| Broad Whitefish \| *Coregonus nasus* \| muscle \| 15N \| 26 \| 10 \| 101.9 \| Hesslein et al. 1993 \| \| Broad Whitefish \| *Coregonus nasus* \| muscle \| 34S \| 26 \| 10 \| 110.0 \| Hesslein et al. 1993 \| \| Brown Shrimp \| *Penaeus aztecus* \| whole organism \| 13C \| 0.00148 \| 24 \| 10.0 \| Fry and Arnold 1982 \| \| Brown Shrimp \| *Penaeus aztecus* \| whole organism \| 13C \| 0.00115 \| 24 \| 19.0 \| Fry and Arnold 1982 \| \| Brown Shrimp \| *Penaeus aztecus* \| whole organism \| 13C \| 0.00148 \| 24 \| 16.0 \| Fry and Arnold 1982 \| \| Brown Shrimp \| *Penaeus aztecus* \| whole organism \| 13C \| 0.00115 \| 24 \| 18.0 \| Fry and Arnold 1982 \| \| Brown Shrimp \| *Penaeus aztecus* \| whole organism \| 13C \| 0.009 \| 24 \| 4.0 \| Fry and Arnold 1982 \| \| Channel Catfish \| *Ictalurus punctatus* \| muscle \| 13C \| 86.7 \| 15 \| 173.3 \| MacAvoy et al. 2001 \| \| Channel Catfish \| *Ictalurus punctatus* \| muscle \| 34S \| 86.7 \| 15 \| 173.3 \| MacAvoy et al. 2001 \| \| Chicken \| *Gallus gallus* \| liver \| 13C \| 48 \|  \| 2.3 \| Cruz et al. 2005 \| \| Chicken \| *Gallus gallus* \| liver \| 13C \| 48 \|  \| 2.8 \| Cruz et al. 2005 \| \| Chicken \| *Gallus gallus* \| liver \| 13C \| 48 \|  \| 4.6 \| Cruz et al. 2005 \| \| Chicken \| *Gallus gallus* \| liver \| 13C \| 48 \|  \| 2.6 \| Cruz et al. 2005 \| \| Chicken \| *Gallus gallus* \| liver \| 13C \| 48 \|  \| 2.1 \| Cruz et al. 2005 \| \| Chicken \| *Gallus gallus* \| liver \| 13C \| 48 \|  \| 1.6 \| Cruz et al. 2005 \| \| Chicken \| *Gallus gallus* \| muscle \| 13C \| 48 \|  \| 2.4 \| Cruz et al. 2005 \| \| Chicken \| *Gallus gallus* \| muscle \| 13C \| 48 \|  \| 5.0 \| Cruz et al. 2005 \| \| Chicken \| *Gallus gallus* \| muscle \| 13C \| 48 \|  \| 10.0 \| Cruz et al. 2005 \| \| Chicken \| *Gallus gallus* \| muscle \| 13C \| 48 \|  \| 7.9 \| Cruz et al. 2005 \| \| Chicken \| *Gallus gallus* \| muscle \| 13C \| 48 \|  \| 8.6 \| Cruz et al. 2005 \| \| Chicken \| *Gallus gallus* \| muscle \| 13C \| 48 \|  \| 2.6 \| Cruz et al. 2005 \| \| Collared Lizard \| *Crotaphytus callaris* \| red blood cells \| 13C \| 47 \| 36 \| 215.9 \| Warne et al. 2010 \| \| Collared Lizard \| *Crotaphytus collaris* \| plasma \| 13C \| 47 \| 36 \| 30.8 \| Warne et al. 2010 \| \| Corn Snake \| *Elaphe guttata* \| blood \| 13C \| 6.2 \| 25 \| 75.0 \| Fisk et al. 2009 \| \| Corn Snake \| *Elaphe guttata* \| blood \| 13C \| 6.2 \| 25 \| 24.0 \| Fisk et al. 2009 \| \| Corn Snake \| *Elaphe guttata* \| liver \| 13C \| 6.2 \| 25 \| 47.0 \| Fisk et al. 2009 \| \| Corn Snake \| *Elaphe guttata* \| liver \| 13C \| 6.2 \| 25 \| 19.0 \| Fisk et al. 2009 \| \| Corn Snake \| *Elaphe guttata* \| muscle \| 13C \| 6.2 \| 25 \| 66.0 \| Fisk et al. 2009 \| \| Cow \| *Bos taurus* \| muscle \| 13C \| 493000 \|  \| 151.0 \| Bahar et al. 2009 \| \| Cow \| *Bos taurus* \| muscle \| 15N \| 493000 \|  \| 157.0 \| Bahar et al. 2009 \| \| Cow \| *Bos taurus* \| muscle \| 34S \| 493000 \|  \| 219.0 \| Bahar et al. 2009 \| \| Deer Mouse \| *Peromyscus maniculatus* \| blood \| 13C \| 18.8 \| 22 \| 18.7 \| Miller et al. 2008 \| \| Deer Mouse \| *Peromyscus maniculatus* \| blood \| 15N \| 18.8 \| 22 \| 19.8 \| Miller et al. 2008 \| \| Deer Mouse \| *Peromyscus maniculatus* \| liver \| 13C \| 18.8 \| 22 \| 2.8 \| Miller et al. 2008 \| \| Deer Mouse \| *Peromyscus maniculatus* \| liver \| 15N \| 18.8 \| 22 \| 3.6 \| Miller et al. 2008 \| \| Deer Mouse \| *Peromyscus maniculatus* \| muscle \| 13C \| 18.8 \| 22 \| 18.7 \| Miller et al. 2008 \| \| Deer Mouse \| *Peromyscus maniculatus* \| muscle \| 15N \| 18.8 \| 22 \| 24.8 \| Miller et al. 2008 \| \| Desert Box Turtle \| *Terrapene ornate luteola* \| red blood cells \| 13C \| 40 \| 30 \| 69.0 \| Murray & Wolf 2013 \| \| Desert Box Turtle \| *Terrapene ornate luteola* \| plasma \| 13C \| 40 \| 30 \| 34.0 \| Murray & Wolf 2013 \| \| Desert Box Turtle \| *Terrapene ornate luteola* \| plasma \| 13C \| 150 \| 30 \| 61.0 \| Murray & Wolf 2013 \| \| Desert Tortoise \| *Gopherus agassizii* \| red blood cells \| 13C \| 64 \| 32 \| 87.8 \| Murray & Wolf 2012 \| \| Desert Tortoise \| *Gopherus agassizii* \| plasma \| 13C \| 64 \| 32 \| 22.8 \| Murray & Wolf 2012 \| \| Dunlin \| *Calidris alpina pacifica* \| blood \| 13C \| 51.7 \|  \| 11.2 \| Evans Ogden et al. 2004 \| \| Dunlin \| *Calidris alpina pacifica* \| blood \| 15N \| 51.7 \|  \| 10.0 \| Evans Ogden et al. 2004 \| \| Earthworm \| *Lumbricus festivus* \| whole organism \| 13C \| 0.81 \| 15 \| 101.0 \| Schmidt et al. 1999 \| \| Earthworm \| *Lumbricus festivus* \| whole organism \| 13C \| 0.81 \| 15 \| 154.0 \| Schmidt et al. 1999 \| \| European Sea Bass \| *Dicentrarchus labrax* \| heart \| 13C \| 8 \| 10 \| 93.7 \| Sweeting et al. 2005 \| \| European Sea Bass \| *Dicentrarchus labrax* \| heart \| 15N \| 8 \| 10 \| 13.7 \| Sweeting et al. 2005 \| \| European Sea Bass \| *Dicentrarchus labrax* \| heart \| 15N \| 8 \| 10 \| 34.3 \| Sweeting et al. 2005 \| \| European Sea Bass \| *Dicentrarchus labrax* \| liver \| 13C \| 8 \| 10 \| 33.5 \| Sweeting et al. 2005 \| \| European Sea Bass \| *Dicentrarchus labrax* \| liver \| 15N \| 8 \| 10 \| 35.9 \| Sweeting et al. 2005 \| \| European Sea Bass \| *Dicentrarchus labrax* \| liver \| 15N \| 8 \| 10 \| 35.4 \| Sweeting et al. 2005 \| \| European Sea Bass \| *Dicentrarchus labrax* \| muscle \| 13C \| 8 \| 10 \| 157.5 \| Sweeting et al. 2005 \| \| European Sea Bass \| *Dicentrarchus labrax* \| muscle \| 15N \| 8 \| 10 \| 49.5 \| Sweeting et al. 2005 \| \| European Sea Bass \| *Dicentrarchus labrax* \| muscle \| 15N \| 8 \| 10 \| 32.2 \| Sweeting et al. 2005 \| \| Florida Manatee \| *Trichechus manatus* \| epidermis \| 13C \| 252000 \|  \| 53.0 \| Alves-Stanley & Worthy 2009 \| \| Florida Manatee \| *Trichechus manatus* \| epidermis \| 13C \| 252000 \|  \| 59.0 \| Alves-Stanley & Worthy 2009 \| \| Florida Manatee \| *Trichechus manatus* \| epidermis \| 15N \| 252000 \|  \| 27.0 \| Alves-Stanley & Worthy 2009 \| \| Florida Manatee \| *Trichechus manatus* \| epidermis \| 15N \| 252000 \|  \| 58.0 \| Alves-Stanley & Worthy 2009 \| \| Gag \| *Mycteroperca microlepis* \| muscle \| 13C \| 7450 \| 22 \| 138.6 \| Nelson et al. 2011 \| \| Garden Warbler \| *Sylvia borin* \| blood \| 13C \| 18.7 \|  \| 5.0 \| Hobson & Bairlein 2003 \| \| Garden Warbler \| *Sylvia borin* \| blood \| 13C \| 18.7 \|  \| 5.7 \| Hobson & Bairlein 2003 \| \| Garden Warbler \| *Sylvia borin* \| blood \| 15N \| 18.7 \|  \| 11.0 \| Hobson & Bairlein 2003 \| \| Garden Warbler \| *Sylvia borin* \| blood \| 15N \| 18.7 \|  \| 5.0 \| Hobson & Bairlein 2003 \| \| Gerbil \| *Meriones unguiculatus* \| brain \| 13C \| 30 \| 25 \| 28.2 \| Tieszen et al. 1983 \| \| Gerbil \| *Meriones unguiculatus* \| liver \| 13C \| 30 \| 25 \| 6.4 \| Tieszen et al. 1983 \| \| Gerbil \| *Meriones unguiculatus* \| muscle \| 13C \| 30 \| 25 \| 28.2 \| Tieszen et al. 1983 \| \| Grass Carp \| *Ctenopharyngodon idella* \| liver \| 13C \| 50 \| 20 \| 22.5 \| Xia et al. 2013 \| \| Grass Carp \| *Ctenopharyngodon idella* \| muscle \| 13C \| 50 \| 20 \| 52.7 \| Xia et al. 2013 \| \| Great Skua \| *Catharacta skua* \| blood \| 13C \| 1218 \|  \| 15.7 \| Bearhop et al. 2002 \| \| Great Skua \| *Catharacta skua* \| blood \| 15N \| 1218 \|  \| 14.4 \| Bearhop et al. 2002 \| \| House Sparrow \| *Passer domesticus* \| red blood cells \| 13C \| 25.17 \| 5 \| 14.7 \| Carleton and Martinez del Rio 2005 \| \| House Sparrow \| *Passer domesticus* \| red blood cells \| 13C \| 25.17 \| 22 \| 16.5 \| Carleton and Martinez del Rio 2005 \| \| House Sparrow \| *Passer domesticus* \| red blood cells \| 15N \| 25.17 \| 5 \| 23.4 \| Carleton and Martinez del Rio 2005 \| \| House Sparrow \| *Passer domesticus* \| red blood cells \| 15N \| 25.17 \| 22 \| 24.6 \| Carleton and Martinez del Rio 2005 \| \| House Sparrow \| *Passer domesticus* \| gizzard \| 13C \| 22 \| 21 \| 14.1 \| Carleton et al. 2008 \| \| House Sparrow \| *Passer domesticus* \| intestine \| 13C \| 22 \| 21 \| 10.3 \| Carleton et al. 2008 \| \| House Sparrow \| *Passer domesticus* \| heart \| 13C \| 22 \| 21 \| 15.2 \| Carleton et al. 2008 \| \| House Sparrow \| *Passer domesticus* \| liver \| 13C \| 22 \| 21 \| 9.8 \| Carleton et al. 2008 \| \| House Sparrow \| *Passer domesticus* \| flight muscle \| 13C \| 22 \| 21 \| 23.5 \| Carleton et al. 2008 \| \| House Sparrow \| *Passer domesticus* \| plasma \| 13C \| 22 \| 21 \| 6.7 \| Carleton et al. 2008 \| \| House Sparrow \| *Passer domesticus* \| red blood cells \| 13C \| 22 \| 21 \| 19.3 \| Carleton et al. 2008 \| \| Japanese Flounder \| *Paralichthys olivaceus* \| muscle \| 13C \| 0.26 \| 17 \| 17.3 \| Tominaga et al. 2003 \| \| Japanese Flounder \| *Paralichthys olivaceus* \| muscle \| 13C \| 0.26 \| 17 \| 13.9 \| Tominaga et al. 2003 \| \| Japanese Flounder \| *Paralichthys olivaceus* \| muscle \| 13C \| 1.06 \| 18.5 \| 5.0 \| Tominaga et al. 2003 \| \| Japanese Quail \| *Coturnix japonica* \| blood \| 13C \| 202 \|  \| 11.4 \| Hobson & Clark 1992 \| \| Japanese Quail \| *Coturnix japonica* \| liver \| 13C \| 202 \|  \| 2.6 \| Hobson & Clark 1992 \| \| Japanese Quail \| *Coturnix japonica* \| muscle \| 13C \| 202 \|  \| 10.0 \| Hobson & Clark 1992 \| \| Japanese Temperate Bass \| *Lateolabrax japonicus* \| liver \| 13C \| 9.87 \| 23 \| 5.3 \| Suzuki et al. 2005 \| \| Japanese Temperate Bass \| *Lateolabrax japonicus* \| liver \| 15N \| 9.87 \| 23 \| 14.4 \| Suzuki et al. 2005 \| \| Japanese Temperate Bass \| *Lateolabrax japonicus* \| muscle \| 13C \| 9.87 \| 23 \| 21.0 \| Suzuki et al. 2005 \| \| Japanese Temperate Bass \| *Lateolabrax japonicus* \| muscle \| 15N \| 9.87 \| 23 \| 19.3 \| Suzuki et al. 2005 \| \| Keeled Slug \| *Milacidae spp.* \| whole organism \| 15N \| 0.426 \| 17 \| 4.3 \| Hakvoort & Schmidt 2002 \| \| Largemouth Bass \| *Micropterus salmoides* \| muscle \| 13C \| 0.52 \| 21.5 \| 17.5 \| Weidel et al. 2011 \| \| Largemouth Bass \| *Micropterus salmoides* \| muscle \| 13C \| 5.6 \| 21.5 \| 24.8 \| Weidel et al. 2011 \| \| Largemouth Bass \| *Micropterus salmoides* \| muscle \| 13C \| 413 \| 21.5 \| 173.3 \| Weidel et al. 2011 \| \| Leopard Shark \| *Triakis semifasciata* \| red blood cells \| 13C \| 3100 \| 15 \| 104.2 \| Kim et al. 2012 \| \| Leopard Shark \| *Triakis semifasciata* \| red blood cells \| 15N \| 3100 \| 15 \| 68.0 \| Kim et al. 2012 \| \| Leopard Shark \| *Triakis semifasciata* \| muscle \| 13C \| 3100 \| 15 \| 225.8 \| Kim et al. 2012 \| \| Leopard Shark \| *Triakis semifasciata* \| muscle \| 15N \| 3100 \| 15 \| 179.6 \| Kim et al. 2012 \| \| Leopard Shark \| *Triakis semifasciata* \| plasma \| 13C \| 3100 \| 15 \| 32.2 \| Kim et al. 2012 \| \| Leopard Shark \| *Triakis semifasciata* \| plasma \| 15N \| 3100 \| 15 \| 42.0 \| Kim et al. 2012 \| \| Leopard Shark \| *Triakis semifasciata* \| muscle \| 13C \| 63 \| 19.3 \| 61.0 \| Malpica-Cruz et al. 2012 \| \| Leopard Shark \| *Triakis semifasciata* \| muscle \| 13C \| 464 \| 19.3 \| 72.0 \| Malpica-Cruz et al. 2012 \| \| Leopard Shark \| *Triakis semifasciata* \| muscle \| 13C \| 1944 \| 19.3 \| 73.0 \| Malpica-Cruz et al. 2012 \| \| Leopard Shark \| *Triakis semifasciata* \| muscle \| 15N \| 63 \| 19.3 \| 91.0 \| Malpica-Cruz et al. 2012 \| \| Leopard Shark \| *Triakis semifasciata* \| muscle \| 15N \| 464 \| 19.3 \| 118.0 \| Malpica-Cruz et al. 2012 \| \| Leopard Shark \| *Triakis semifasciata* \| muscle \| 15N \| 1944 \| 19.3 \| 122.0 \| Malpica-Cruz et al. 2012 \| \| Limacid Slug \| *Deroceras reticulatum* \| whole organism \| 15N \| 0.322 \| 17 \| 19.3 \| Hakvoort & Schmidt 2002 \| \| Loggerhead Turtle \| *Caretta caretta* \| blood \| 13C \| 19.8 \| 26.5 \| 30.2 \| Reich et al. 2008 \| \| Loggerhead Turtle \| *Caretta caretta* \| blood \| 15N \| 19.8 \| 26.5 \| 24.8 \| Reich et al. 2008 \| \| Loggerhead Turtle \| *Caretta caretta* \| red blood cells \| 13C \| 19.8 \| 26.5 \| 53.3 \| Reich et al. 2008 \| \| Loggerhead Turtle \| *Caretta caretta* \| red blood cells \| 15N \| 19.8 \| 26.5 \| 49.5 \| Reich et al. 2008 \| \| Loggerhead Turtle \| *Caretta caretta* \| plasma \| 13C \| 19.8 \| 26.5 \| 13.9 \| Reich et al. 2008 \| \| Loggerhead Turtle \| *Caretta caretta* \| plasma \| 15N \| 19.8 \| 26.5 \| 12.8 \| Reich et al. 2008 \| \| Long-Nosed Bandicoot \| *Parameles nasuta* \| blood cells \| 13C \| 1150 \| 6 \| 90.0 \| Klaassen et al. 2004 \| \| Long-Nosed Bandicoot \| *Parameles nasuta* \| blood cells \| 15N \| 1150 \| 6 \| 90.0 \| Klaassen et al. 2004 \| \| Long-Nosed Bandicoot \| *Parameles nasuta* \| blood plasma \| 13C \| 1150 \| 6 \| 9.0 \| Klaassen et al. 2004 \| \| Long-Nosed Bandicoot \| *Parameles nasuta* \| blood plasma \| 15N \| 1150 \| 6 \| 9.0 \| Klaassen et al. 2004 \| \| Migratory Goby \| *Rhinogobius spp.* \| muscle \| 15N \| 0.109 \|  \| 33.0 \| Maruyama et al. 2001 \| \| Migratory Goby \| *Rhinogobius spp.* \| muscle \| 15N \| 0.109 \|  \| 99.0 \| Maruyama et al. 2001 \| \| Mouse \| *Mus musculus* \| blood \| 13C \| 28.7 \|  \| 17.3 \| MacAvoy et al. 2006 \| \| Mouse \| *Mus musculus* \| blood \| 13C \| 17.9 \|  \| 16.9 \| MacAvoy et al. 2005 \| \| Mouse \| *Mus musculus* \| blood \| 13C \| 20.42 \|  \| 19.8 \| Arneson et al. 2006 \| \| Mouse \| *Mus musculus* \| blood \| 13C \| 20.51 \|  \| 20.4 \| Arneson et al. 2006 \| \| Mouse \| *Mus musculus* \| blood \| 15N \| 28.7 \|  \| 15.4 \| MacAvoy et al. 2006 \| \| Mouse \| *Mus musculus* \| blood \| 15N \| 17.9 \|  \| 19.3 \| MacAvoy et al. 2005 \| \| Mouse \| *Mus musculus* \| blood \| 15N \| 20.42 \|  \| 15.4 \| Arneson et al. 2006 \| \| Mouse \| *Mus musculus* \| blood \| 15N \| 20.42 \|  \| 23.9 \| Arneson et al. 2006 \| \| Mouse \| *Mus musculus* \| blood \| 15N \| 20.51 \|  \| 23.9 \| Arneson et al. 2006 \| \| Mouse \| *Mus musculus* \| blood \| 34S \| 20.42 \|  \| 12.6 \| Arneson et al. 2006 \| \| Mouse \| *Mus musculus* \| blood \| 34S \| 20.42 \|  \| 9.9 \| Arneson et al. 2006 \| \| Mouse \| *Mus musculus* \| blood \| 34S \| 20.51 \|  \| 20.4 \| Arneson et al. 2006 \| \| Mouse \| *Mus musculus* \| blood \| 34S \| 20.51 \|  \| 10.3 \| Arneson et al. 2006 \| \| Mouse \| *Mus musculus* \| brain \| 13C \| 20.51 \|  \| 17.8 \| Arneson et al. 2006 \| \| Mouse \| *Mus musculus* \| heart \| 13C \| 20.51 \|  \| 13.9 \| Arneson et al. 2006 \| \| Mouse \| *Mus musculus* \| kidney \| 13C \| 20.51 \|  \| 4.6 \| Arneson et al. 2006 \| \| Mouse \| *Mus musculus* \| liver \| 13C \| 17.9 \|  \| 46.2 \| MacAvoy et al. 2005 \| \| Mouse \| *Mus musculus* \| liver \| 13C \| 20.42 \|  \| 7.7 \| Arneson et al. 2006 \| \| Mouse \| *Mus musculus* \| liver \| 13C \| 20.51 \|  \| 5.0 \| Arneson et al. 2006 \| \| Mouse \| *Mus musculus* \| brain \| 15N \| 20.51 \|  \| 22.4 \| Arneson et al. 2006 \| \| Mouse \| *Mus musculus* \| heart \| 15N \| 20.51 \|  \| 19.3 \| Arneson et al. 2006 \| \| Mouse \| *Mus musculus* \| heart \| 15N \| 20.51 \|  \| 15.8 \| Arneson et al. 2006 \| \| Mouse \| *Mus musculus* \| kidney \| 15N \| 20.51 \|  \| 21.7 \| Arneson et al. 2006 \| \| Mouse \| *Mus musculus* \| kidney \| 15N \| 20.51 \|  \| 10.0 \| Arneson et al. 2006 \| \| Mouse \| *Mus musculus* \| liver \| 15N \| 17.9 \|  \| 7.3 \| MacAvoy et al. 2005 \| \| Mouse \| *Mus musculus* \| liver \| 15N \| 20.42 \|  \| 15.1 \| Arneson et al. 2006 \| \| Mouse \| *Mus musculus* \| liver \| 15N \| 20.42 \|  \| 12.0 \| Arneson et al. 2006 \| \| Mouse \| *Mus musculus* \| liver \| 15N \| 20.51 \|  \| 6.9 \| Arneson et al. 2006 \| \| Mouse \| *Mus musculus* \| liver \| 34S \| 20.42 \|  \| 8.3 \| Arneson et al. 2006 \| \| Mouse \| *Mus musculus* \| liver \| 34S \| 20.42 \|  \| 9.1 \| Arneson et al. 2006 \| \| Mouse \| *Mus musculus* \| liver \| 34S \| 20.51 \|  \| 7.1 \| Arneson et al. 2006 \| \| Mouse \| *Mus musculus* \| liver \| 34S \| 20.51 \|  \| 3.9 \| Arneson et al. 2006 \| \| Mouse \| *Mus musculus* \| muscle \| 13C \| 17.9 \|  \| 23.9 \| MacAvoy et al. 2005 \| \| Mouse \| *Mus musculus* \| muscle \| 13C \| 20.42 \|  \| 18.2 \| Arneson et al. 2006 \| \| Mouse \| *Mus musculus* \| muscle \| 13C \| 20.51 \|  \| 23.1 \| Arneson et al. 2006 \| \| Mouse \| *Mus musculus* \| muscle \| 15N \| 17.9 \|  \| 24.8 \| MacAvoy et al. 2005 \| \| Mouse \| *Mus musculus* \| muscle \| 15N \| 20.42 \|  \| 18.2 \| Arneson et al. 2006 \| \| Mouse \| *Mus musculus* \| muscle \| 15N \| 20.42 \|  \| 21.7 \| Arneson et al. 2006 \| \| Mouse \| *Mus musculus* \| muscle \| 15N \| 20.51 \|  \| 46.2 \| Arneson et al. 2006 \| \| Mouse \| *Mus musculus* \| muscle \| 15N \| 20.51 \|  \| 15.8 \| Arneson et al. 2006 \| \| Mouse \| *Mus musculus* \| muscle \| 34S \| 20.42 \|  \| 21.4 \| Arneson et al. 2006 \| \| Mouse \| *Mus musculus* \| muscle \| 34S \| 20.42 \|  \| 34.7 \| Arneson et al. 2006 \| \| Mouse \| *Mus musculus* \| muscle \| 34S \| 20.51 \|  \| 33.0 \| Arneson et al. 2006 \| \| Mouse \| *Mus musculus* \| muscle \| 34S \| 20.51 \|  \| 14.4 \| Arneson et al. 2006 \| \| Mouse \| *Mus musculus* \| blood \| 13C \| 31.9 \|  \| 11.1 \| MacAvoy et al. 2012 \| \| Mouse \| *Mus musculus* \| blood \| 13C \| 23.1 \|  \| 10.6 \| MacAvoy et al. 2012 \| \| Mouse \| *Mus musculus* \| blood \| 15N \| 31.9 \|  \| 30.0 \| MacAvoy et al. 2012 \| \| Mouse \| *Mus musculus* \| blood \| 15N \| 23.1 \|  \| 17.4 \| MacAvoy et al. 2012 \| \| Natterjack Toad \| *Bufo calamita* \| muscle \| 13C \| 1 \| 20 \| 9.2 \| Caut et al. 2013 \| \| Natterjack Toad \| *Bufo calamita* \| muscle \| 15N \| 1 \| 20 \| 16.4 \| Caut et al. 2013 \| \| Natterjack Toad \| *Bufo calamita* \| muscle \| 15N \| 1 \| 20 \| 24.0 \| Caut et al. 2013 \| \| Natterjack Toad \| *Bufo calamita* \| muscle \| 15N \| 1 \| 20 \| 14.5 \| Caut et al. 2013 \| \| Natterjack Toad \| *Bufo calamita* \| muscle \| 15N \| 1 \| 20 \| 12.6 \| Caut et al. 2013 \| \| Natterjack Toad \| *Bufo calamita* \| muscle \| 15N \| 1 \| 20 \| 17.7 \| Caut et al. 2013 \| \| Natterjack Toad \| *Bufo calamita* \| muscle \| 15N \| 1 \| 20 \| 15.5 \| Caut et al. 2013 \| \| Nile tilapia \| *Oreochromis niloticus* \| muscle \| 13C \| 24.46 \| 25 \| 23.3 \| Zuanon et al. 2007 \| \| Nile tilapia \| *Oreochromis niloticus* \| muscle \| 13C \| 23.78 \| 25 \| 26.0 \| Zuanon et al. 2007 \| \| Nile tilapia \| *Oreochromis niloticus* \| liver \| 13C \| 4.40 \| 26 \| 83.7 \| Carleton & Martinez del Rio 2010 \| \| Nile tilapia \| *Oreochromis niloticus* \| liver \| 13C \| 4.40 \| 26 \| 13.3 \| Carleton & Martinez del Rio 2010 \| \| Nile tilapia \| *Oreochromis niloticus* \| liver \| 13C \| 4.40 \| 26 \| 16.2 \| Carleton & Martinez del Rio 2010 \| \| Nile tilapia \| *Oreochromis niloticus* \| muscle \| 13C \| 4.40 \| 26 \| 142.7 \| Carleton & Martinez del Rio 2010 \| \| Nile tilapia \| *Oreochromis niloticus* \| muscle \| 13C \| 4.40 \| 26 \| 37.9 \| Carleton & Martinez del Rio 2010 \| \| Nile tilapia \| *Oreochromis niloticus* \| muscle \| 13C \| 4.40 \| 26 \| 64.5 \| Carleton & Martinez del Rio 2010 \| \| Ocellate River Stingray \| *Potamotrygon motoro* \| blood \| 15N \| 106.37 \| 26 \| 61.0 \| MacNeil et al. 2006 \| \| Ocellate River Stingray \| *Potamotrygon motoro* \| blood \| 15N \| 106.46 \| 26 \| 53.0 \| MacNeil et al. 2006 \| \| Ocellate River Stingray \| *Potamotrygon motoro* \| liver \| 15N \| 106.46 \| 26 \| 14.0 \| MacNeil et al. 2006 \| \| Ocellate River Stingray \| *Potamotrygon motoro* \| liver \| 15N \| 106.37 \| 26 \| 39.0 \| MacNeil et al. 2006 \| \| Ocellate River Stingray \| *Potamotrygon motoro* \| muscle \| 15N \| 106.37 \| 26 \| 98.0 \| MacNeil et al. 2006 \| \| Pacific Bluefin Tuna \| *Thunnus orientalis* \| liver \| 13C \| 8440 \| 20 \| 162.0 \| Madigan et al. 2012 \| \| Pacific Bluefin Tuna \| *Thunnus orientalis* \| liver \| 15N \| 8440 \| 20 \| 86.0 \| Madigan et al. 2012 \| \| Pacific Bluefin Tuna \| *Thunnus orientalis* \| muscle \| 13C \| 8440 \| 20 \| 255.0 \| Madigan et al. 2012 \| \| Pacific Bluefin Tuna \| *Thunnus orientalis* \| muscle \| 15N \| 8440 \| 20 \| 167.0 \| Madigan et al. 2012 \| \| Pacific Herring \| *Clupea pallasi* \| blood \| 15N \| 47 \| 10.6 \| 30.1 \| Miller 2000 \| \| Pacific Herring \| *Clupea pallasi* \| heart \| 15N \| 47 \| 10.6 \| 31.5 \| Miller 2000 \| \| Pacific Herring \| *Clupea pallasi* \| liver \| 15N \| 47 \| 10.6 \| 21.0 \| Miller 2000 \| \| Pacific Herring \| *Clupea pallasi* \| muscle \| 15N \| 47 \| 10.6 \| 46.2 \| Miller 2000 \| \| Pacific Oyster \| *Crassostrea gigas* \| muscle \| 13C \| 0.25 \| 15.9 \| 7.7 \| Dubois et al. 2007 \| \| Pacific Oyster \| *Crassostrea gigas* \| muscle \| 15N \| 0.25 \| 15.9 \| 15.1 \| Dubois et al. 2007 \| \| Pallas' Long-Tongued Bat \| *Glossophaga soricina* \| blood \| 13C \| 10.2 \| 23 \| 113.0 \| Voigt et al. 2003 \| \| Pallas' Long-Tongued Bat \| *Glossophaga soricina* \| blood \| 13C \| 10.2 \| 26 \| 24.2 \| Miron et al. 2006 \| \| Pallas' Long-Tongued Bat \| *Glossophaga soricina* \| blood \| 13C \| 10.2 \| 26 \| 39.6 \| Miron et al. 2006 \| \| Pallas' Long-Tongued Bat \| *Glossophaga soricina* \| blood \| 15N \| 10.2 \| 26 \| 25.5 \| Miron et al. 2006 \| \| Pallas' Long-Tongued Bat \| *Glossophaga soricina* \| blood \| 15N \| 10.2 \| 26 \| 24.9 \| Miron et al. 2006 \| \| Pintado \| *Pseudoplatystoma corruscans* \| muscle \| 13C \| 0.77 \| 26.69 \| 4.4 \| Furuya et al. 2002 \| \| Pirate Bug \| *Orius majusculus* \| whole organism \| 13C \| 0.005 \| 24 \| 2.2 \| Madeira et al. 2013 \| \| Pond Slider \| *Trachemys scripta* \| blood \| 15N \| 842 \| 26 \| 38.7 \| Seminoff et al. 2007 \| \| Pond Slider \| *Trachemys scripta* \| liver \| 15N \| 842 \| 26 \| 52.5 \| Seminoff et al. 2007 \| \| Pond Slider \| *Trachemys scripta* \| blood plasma \| 13C \| 842 \| 26 \| 42.5 \| Seminoff et al. 2007 \| \| Pond Slider \| *Trachemys scripta* \| blood plasma \| 15N \| 842 \| 26 \| 35.6 \| Seminoff et al. 2007 \| \| Prairie Lizard \| *Sceloporus undulatus* \| red blood cells \| 13C \| 12 \| 36 \| 42.1 \| Warne et al. 2010 \| \| Prairie Lizard \| *Sceloporus undulatus* \| liver \| 13C \| 12 \| 36 \| 14.8 \| Warne et al. 2010 \| \| Prairie Lizard \| *Sceloporus undulatus* \| muscle \| 13C \| 12 \| 36 \| 56.1 \| Warne et al. 2010 \| \| Prairie Lizard \| *Sceloporus undulatus* \| plasma \| 13C \| 12 \| 36 \| 17.3 \| Warne et al. 2010 \| \| Quilted Melania \| *Tarebia granifera* \| muscle \| 15N \| 0.08 \| 23.5 \| 20.2 \| McIntyre & Flecker 2006 \| \| Ragworm \| *Nereis virens* \| whole organism \| 13C \| 0.25 \| 15 \| 17.3 \| Olive et al. 2003 \| \| Ragworm \| *Nereis virens* \| whole organism \| 15N \| 0.25 \| 15 \| 9.9 \| Olive et al. 2003 \| \| Rainbow Trout \| *Oncorhynchus mykiss* \| red blood cells \| 15N \| 0.48 \| 14 \| 26.1 \| Heady & Moore 2013 \| \| Rainbow Trout \| *Oncorhynchus mykiss* \| liver \| 15N \| 0.48 \| 14 \| 11.2 \| Heady & Moore 2013 \| \| Rainbow Trout \| *Oncorhynchus mykiss* \| muscle \| 15N \| 0.48 \| 14 \| 27.0 \| Heady & Moore 2013 \| \| Rainbow Trout \| *Oncorhynchus mykiss* \| plasma \| 15N \| 0.48 \| 14 \| 9.8 \| Heady & Moore 2013 \| \| Rat \| *Rattus norvegicus* \| blood \| 13C \| 279.2 \|  \| 24.8 \| MacAvoy et al. 2006 \| \| Rat \| *Rattus norvegicus* \| blood \| 15N \| 279.2 \|  \| 27.7 \| MacAvoy et al. 2006 \| \| Rat \| *Rattus norvegicus* \| brain \| 13C \| 50 \| 25 \| 19.0 \| Braun et al. 2013 \| \| Rat \| *Rattus norvegicus* \| brain \| 13C \| 50 \| 25 \| 14.0 \| Braun et al. 2013 \| \| Rat \| *Rattus norvegicus* \| brain \| 15N \| 50 \| 25 \| 16.0 \| Braun et al. 2013 \| \| Rat \| *Rattus norvegicus* \| brain \| 15N \| 50 \| 25 \| 21.0 \| Braun et al. 2013 \| \| Rat \| *Rattus norvegicus* \| heart \| 13C \| 50 \| 25 \| 30.0 \| Braun et al. 2013 \| \| Rat \| *Rattus norvegicus* \| heart \| 13C \| 50 \| 25 \| 25.0 \| Braun et al. 2013 \| \| Rat \| *Rattus norvegicus* \| heart \| 15N \| 50 \| 25 \| 24.0 \| Braun et al. 2013 \| \| Rat \| *Rattus norvegicus* \| heart \| 15N \| 50 \| 25 \| 32.0 \| Braun et al. 2013 \| \| Rat \| *Rattus norvegicus* \| kidney \| 13C \| 50 \| 25 \| 8.0 \| Braun et al. 2013 \| \| Rat \| *Rattus norvegicus* \| kidney \| 13C \| 50 \| 25 \| 7.0 \| Braun et al. 2013 \| \| Rat \| *Rattus norvegicus* \| kidney \| 15N \| 50 \| 25 \| 10.0 \| Braun et al. 2013 \| \| Rat \| *Rattus norvegicus* \| kidney \| 15N \| 50 \| 25 \| 6.0 \| Braun et al. 2013 \| \| Rat \| *Rattus norvegicus* \| liver \| 13C \| 50 \| 25 \| 10.0 \| Braun et al. 2013 \| \| Rat \| *Rattus norvegicus* \| liver \| 13C \| 50 \| 25 \| 6.0 \| Braun et al. 2013 \| \| Rat \| *Rattus norvegicus* \| liver \| 15N \| 50 \| 25 \| 10.0 \| Braun et al. 2013 \| \| Rat \| *Rattus norvegicus* \| liver \| 15N \| 50 \| 25 \| 9.0 \| Braun et al. 2013 \| \| Rat \| *Rattus norvegicus* \| lung \| 13C \| 50 \| 25 \| 9.0 \| Braun et al. 2013 \| \| Rat \| *Rattus norvegicus* \| lung \| 13C \| 50 \| 25 \| 11.0 \| Braun et al. 2013 \| \| Rat \| *Rattus norvegicus* \| lung \| 15N \| 50 \| 25 \| 12.0 \| Braun et al. 2013 \| \| Rat \| *Rattus norvegicus* \| lung \| 15N \| 50 \| 25 \| 8.0 \| Braun et al. 2013 \| \| Rat \| *Rattus norvegicus* \| spleen \| 13C \| 50 \| 25 \| 7.0 \| Braun et al. 2013 \| \| Rat \| *Rattus norvegicus* \| spleen \| 13C \| 50 \| 25 \| 6.0 \| Braun et al. 2013 \| \| Rat \| *Rattus norvegicus* \| spleen \| 15N \| 50 \| 25 \| 7.0 \| Braun et al. 2013 \| \| Rat \| *Rattus norvegicus* \| spleen \| 15N \| 50 \| 25 \| 13.0 \| Braun et al. 2013 \| \| Rat \| *Rattus norvegicus* \| muscle \| 13C \| 50 \| 25 \| 38.0 \| Braun et al. 2013 \| \| Rat \| *Rattus norvegicus* \| muscle \| 13C \| 50 \| 25 \| 26.0 \| Braun et al. 2013 \| \| Rat \| *Rattus norvegicus* \| muscle \| 15N \| 50 \| 25 \| 37.0 \| Braun et al. 2013 \| \| Rat \| *Rattus norvegicus* \| muscle \| 15N \| 50 \| 25 \| 45.0 \| Braun et al. 2013 \| \| Rat \| *Rattus norvegicus* \| plasma \| 13C \| 50 \| 25 \| 5.0 \| Braun et al. 2013 \| \| Rat \| *Rattus norvegicus* \| plasma \| 13C \| 50 \| 25 \| 4.0 \| Braun et al. 2013 \| \| Rat \| *Rattus norvegicus* \| plasma \| 15N \| 50 \| 25 \| 8.0 \| Braun et al. 2013 \| \| Rat \| *Rattus norvegicus* \| plasma \| 15N \| 50 \| 25 \| 3.0 \| Braun et al. 2013 \| \| Red Drum \| *Sciaenops ocellatus* \| whole organism \| 13C \| 0.00062 \| 22 \| 8.0 \| Herzka et al. 2001 \| \| Red Drum \| *Sciaenops ocellatus* \| whole organism \| 13C \| 0.00089 \| 24 \| 7.0 \| Herzka & Holt 2000 \| \| Red Drum \| *Sciaenops ocellatus* \| whole organism \| 13C \| 0.000024 \| 28 \| 6.0 \| Herzka & Holt 2000 \| \| Red Drum \| *Sciaenops ocellatus* \| whole organism \| 13C \| 0.00074 \| 28 \| 5.0 \| Herzka & Holt 2000 \| \| Red Drum \| *Sciaenops ocellatus* \| whole organism \| 15N \| 0.00062 \| 22 \| 8.0 \| Herzka et al. 2001 \| \| Red Drum \| *Sciaenops ocellatus* \| whole organism \| 15N \| 0.00089 \| 24 \| 7.0 \| Herzka & Holt 2000 \| \| Red Drum \| *Sciaenops ocellatus* \| whole organism \| 15N \| 0.000024 \| 28 \| 6.0 \| Herzka & Holt 2000 \| \| Red Drum \| *Sciaenops ocellatus* \| whole organism \| 15N \| 0.00074 \| 28 \| 5.0 \| Herzka & Holt 2000 \| \| Red Rock Lobster \| *Jasus edwardsii* \| hemolymph \| 13C \| 153.1 \| 12 \| 116.8 \| Suring & Wing 2009 \| \| Red Rock Lobster \| *Jasus edwardsii* \| muscle \| 13C \| 153.1 \| 12 \| 147.0 \| Suring & Wing 2009 \| \| Sand Goby \| *Pomatoschistus minutus* \| heart \| 13C \| 5.85 \| 17 \| 6.1 \| Guelinckx et al. 2007 \| \| Sand Goby \| *Pomatoschistus minutus* \| liver \| 13C \| 5.85 \| 17 \| 9.1 \| Guelinckx et al. 2007 \| \| Sand Goby \| *Pomatoschistus minutus* \| heart \| 15N \| 5.85 \| 17 \| 27.6 \| Guelinckx et al. 2007 \| \| Sand Goby \| *Pomatoschistus minutus* \| liver \| 15N \| 5.85 \| 17 \| 2.8 \| Guelinckx et al. 2007 \| \| Sand Goby \| *Pomatoschistus minutus* \| muscle \| 13C \| 5.85 \| 17 \| 24.7 \| Guelinckx et al. 2007 \| \| Sand Goby \| *Pomatoschistus minutus* \| muscle \| 15N \| 5.85 \| 17 \| 27.8 \| Guelinckx et al. 2007 \| \| Sandbar Shark \| *Carcharhinus plumbeus* \| blood \| 13C \| 11300 \| 23.5 \| 93.3 \| Logan & Lutcavage 2010 \| \| Sandbar Shark \| *Carcharhinus plumbeus* \| blood \| 15N \| 11300 \| 23.5 \| 60.6 \| Logan & Lutcavage 2010 \| \| Sandbar Shark \| *Carcharhinus plumbeus* \| muscle \| 13C \| 11300 \| 23.5 \| 155.2 \| Logan & Lutcavage 2010 \| \| Sandbar Shark \| *Carcharhinus plumbeus* \| muscle \| 15N \| 11300 \| 23.5 \| 92.4 \| Logan & Lutcavage 2010 \| \| Saussure's Long-Nosed Bat \| *Leptonycteris curasoae* \| blood \| 13C \| 23.61 \| 23 \| 120.0 \| Voigt et al. 2003 \| \| Sea Cucumber \| *Apostichopus japonicus* \| intestine \| 13C \| 5.14 \| 17 \| 11.2 \| Sun et al. 2012 \| \| Sea Cucumber \| *Apostichopus japonicus* \| intestine \| 13C \| 15.3 \| 17 \| 23.5 \| Sun et al. 2012 \| \| Sea Cucumber \| *Apostichopus japonicus* \| intestine \| 13C \| 36.4 \| 17 \| 37.1 \| Sun et al. 2012 \| \| Sea Cucumber \| *Apostichopus japonicus* \| intestine \| 13C \| 78.4 \| 17 \| 53.7 \| Sun et al. 2012 \| \| Sea Cucumber \| *Apostichopus japonicus* \| body wall muscle \| 13C \| 5.14 \| 17 \| 21.4 \| Sun et al. 2012 \| \| Sea Cucumber \| *Apostichopus japonicus* \| body wall muscle \| 13C \| 15.3 \| 17 \| 54.2 \| Sun et al. 2012 \| \| Sea Cucumber \| *Apostichopus japonicus* \| body wall muscle \| 13C \| 36.4 \| 17 \| 65.4 \| Sun et al. 2012 \| \| Sea Cucumber \| *Apostichopus japonicus* \| body wall muscle \| 13C \| 78.4 \| 17 \| 105.0 \| Sun et al. 2012 \| \| Senegalese Sole \| *Solea senegalensis* \| whole organism \| 13C \| 0.0000263 \| 21.5 \| 3.5 \| Gamboa-Delgado et al. 2008 \| \| Senegalese Sole \| *Solea senegalensis* \| whole organism \| 13C \| 0.0000263 \| 21.5 \| 2.7 \| Gamboa-Delgado et al. 2008 \| \| Senegalese Sole \| *Solea senegalensis* \| whole organism \| 13C \| 0.0000263 \| 21.5 \| 2.5 \| Gamboa-Delgado et al. 2008 \| \| Sheep \| *Ovis aries* \| muscle \| 13C \| 5000 \|  \| 55.9 \| Harrison et al. 2011 \| \| Sheep \| *Ovis aries* \| muscle \| 13C \| 5000 \|  \| 55.0 \| Harrison et al. 2011 \| \| Sheep \| *Ovis aries* \| muscle \| 34S \| 5000 \|  \| 53.3 \| Harrison et al. 2011 \| \| Sheep \| *Ovis aries* \| muscle \| 34S \| 5000 \|  \| 57.7 \| Harrison et al. 2011 \| \| Smallmouth Bass \| *Micropterus dolomieu* \| whole organism \| 15N \| 0.000154 \|  \| 15.0 \| Vander Zanden et al. 1998 \| \| Sockeye Salmon \| *Oncorhynchus nerka* \| muscle \| 15N \| 238.4 \| 11 \| 40.8 \| Sakano et al. 2005 \| \| Sockeye Salmon \| *Oncorhynchus nerka* \| muscle \| 15N \| 120.6 \| 11 \| 17.3 \| Sakano et al. 2005 \| \| Sockeye Salmon \| *Oncorhynchus nerka* \| muscle \| 15N \| 12.4 \| 11 \| 14.4 \| Sakano et al. 2005 \| \| Springtail \| *Folsomia candida* \| whole organism \| 13C \| 0.00125 \| 20 \| 7.3 \| Larsen et al. 2011 \| \| Springtail \| *Folsomia candida* \| whole organism \| 13C \| 0.00125 \| 20 \| 8.4 \| Larsen et al. 2011 \| \| Springtail \| *Folsomia candida* \| whole organism \| 13C \| 0.00125 \| 20 \| 4.4 \| Larsen et al. 2011 \| \| Springtail \| *Folsomia candida* \| whole organism \| 13C \| 0.00125 \| 20 \| 4.3 \| Larsen et al. 2011 \| \| Springtail \| *Folsomia candida* \| whole organism \| 15N \| 0.00125 \| 20 \| 8.0 \| Larsen et al. 2011 \| \| Springtail \| *Folsomia candida* \| whole organism \| 15N \| 0.00125 \| 20 \| 9.0 \| Larsen et al. 2011 \| \| Springtail \| *Folsomia candida* \| whole organism \| 15N \| 0.00125 \| 20 \| 4.6 \| Larsen et al. 2011 \| \| Springtail \| *Folsomia candida* \| whole organism \| 15N \| 0.00125 \| 20 \| 3.7 \| Larsen et al. 2011 \| \| Springtail \| *Proisotoma minuta* \| whole organism \| 13C \| 0.00125 \| 20 \| 10.0 \| Larsen et al. 2011 \| \| Springtail \| *Proisotoma minuta* \| whole organism \| 13C \| 0.00125 \| 20 \| 3.8 \| Larsen et al. 2011 \| \| Springtail \| *Proisotoma minuta* \| whole organism \| 15N \| 0.00125 \| 20 \| 9.3 \| Larsen et al. 2011 \| \| Springtail \| *Proisotoma minuta* \| whole organism \| 15N \| 0.00125 \| 20 \| 3.7 \| Larsen et al. 2011 \| \| Springtail \| *Protaphorura fimata* \| whole organism \| 13C \| 0.00125 \| 20 \| 4.3 \| Larsen et al. 2011 \| \| Springtail \| *Protaphorura fimata* \| whole organism \| 13C \| 0.00125 \| 20 \| 4.0 \| Larsen et al. 2011 \| \| Springtail \| *Protaphorura fimata* \| whole organism \| 15N \| 0.00125 \| 20 \| 5.0 \| Larsen et al. 2011 \| \| Springtail \| *Protaphorura fimata* \| whole organism \| 15N \| 0.00125 \| 20 \| 6.4 \| Larsen et al. 2011 \| \| Steelhead \| *Oncorhynchus mykiss* \| muscle \| 13C \| 2.63 \|  \| 136.0 \| Church et al. 2009 \| \| Steelhead \| *Oncorhynchus mykiss* \| muscle \| 15N \| 2.63 \|  \| 94.0 \| Church et al. 2009 \| \| Summer Flounder \| *Paralichthys dentatus* \| whole organism \| 13C \| 0.0001 \| 13 \| 9.2 \| Witting et al. 2004 \| \| Summer Flounder \| *Paralichthys dentatus* \| whole organism \| 13C \| 0.0017 \| 13 \| 11.6 \| Witting et al. 2004 \| \| Summer Flounder \| *Paralichthys dentatus* \| whole organism \| 13C \| 0.0031 \| 13 \| 16.9 \| Witting et al. 2004 \| \| Summer Flounder \| *Paralichthys dentatus* \| whole organism \| 13C \| 0.0001 \| 22 \| 2.9 \| Witting et al. 2004 \| \| Summer Flounder \| *Paralichthys dentatus* \| whole organism \| 13C \| 0.0017 \| 22 \| 5.0 \| Witting et al. 2004 \| \| Summer Flounder \| *Paralichthys dentatus* \| whole organism \| 13C \| 0.0031 \| 22 \| 6.0 \| Witting et al. 2004 \| \| Summer Flounder \| *Paralichthys dentatus* \| whole organism \| 15N \| 0.0001 \| 13 \| 7.5 \| Witting et al. 2004 \| \| Summer Flounder \| *Paralichthys dentatus* \| whole organism \| 15N \| 0.0017 \| 13 \| 13.6 \| Witting et al. 2004 \| \| Summer Flounder \| *Paralichthys dentatus* \| whole organism \| 15N \| 0.0031 \| 13 \| 63.0 \| Witting et al. 2004 \| \| Summer Flounder \| *Paralichthys dentatus* \| whole organism \| 15N \| 0.0001 \| 22 \| 3.2 \| Witting et al. 2004 \| \| Summer Flounder \| *Paralichthys dentatus* \| whole organism \| 15N \| 0.0017 \| 22 \| 6.1 \| Witting et al. 2004 \| \| Summer Flounder \| *Paralichthys dentatus* \| whole organism \| 15N \| 0.0031 \| 22 \| 99.0 \| Witting et al. 2004 \| \| Summer Flounder \| *Paralichthys dentatus* \| blood \| 13C \| 59 \| 20 \| 21.6 \| Buchheister & Latour 2010 \| \| Summer Flounder \| *Paralichthys dentatus* \| blood \| 13C \| 102 \| 20 \| 22.8 \| Buchheister & Latour 2010 \| \| Summer Flounder \| *Paralichthys dentatus* \| blood \| 15N \| 59 \| 20 \| 43.8 \| Buchheister & Latour 2010 \| \| Summer Flounder \| *Paralichthys dentatus* \| blood \| 15N \| 102 \| 20 \| 33 \| Buchheister & Latour 2010 \| \| Summer Flounder \| *Paralichthys dentatus* \| liver \| 13C \| 59 \| 20 \| 20.2 \| Buchheister & Latour 2010 \| \| Summer Flounder \| *Paralichthys dentatus* \| liver \| 13C \| 102 \| 20 \| 16.6 \| Buchheister & Latour 2010 \| \| Summer Flounder \| *Paralichthys dentatus* \| liver \| 15N \| 59 \| 20 \| 9.7 \| Buchheister & Latour 2010 \| \| Summer Flounder \| *Paralichthys dentatus* \| liver \| 15N \| 102 \| 20 \| 9.9 \| Buchheister & Latour 2010 \| \| Sympagic Amphipod \| *Onisimus litoralis* \| whole organism \| 13C \| 0.0549 \| 1 \| 77.0 \| Kaufman et al. 2008 \| \| Sympagic Amphipod \| *Onisimus litoralis* \| whole organism \| 13C \| 0.0519 \| 1 \| 18.7 \| Kaufman et al. 2008 \| \| Sympagic Amphipod \| *Onisimus litoralis* \| whole organism \| 13C \| 0.0546 \| 4 \| 13.9 \| Kaufman et al. 2008 \| \| Sympagic Amphipod \| *Onisimus litoralis* \| whole organism \| 15N \| 0.0556 \| 1 \| 115.5 \| Kaufman et al. 2008 \| \| Sympagic Amphipod \| *Onisimus litoralis* \| whole organism \| 15N \| 0.0544 \| 1 \| 20.4 \| Kaufman et al. 2008 \| \| Sympagic Amphipod \| *Onisimus litoralis* \| whole organism \| 15N \| 0.0519 \| 1 \| 22.4 \| Kaufman et al. 2008 \| \| Sympagic Amphipod \| *Onisimus litoralis* \| whole organism \| 15N \| 0.0546 \| 4 \| 22.4 \| Kaufman et al. 2008 \| \| Tanganyika Snail \| *Lavigeria grandis* \| muscle \| 15N \| 0.78 \| 26 \| 49.5 \| McIntyre & Flecker 2006 \| \| Water Strider \| *Aquarius remigis* \| whole organism \| 13C \| 0.0108 \|  \| 1.5 \| Jardine et al. 2008 \| \| Water Strider \| *Aquarius remigis* \| whole organism \| 15N \| 0.0108 \|  \| 7.8 \| Jardine et al. 2008 \| \| Western Spadefoot Toad \| *Pelobates cultripes* \| mucle \| 13C \| 1 \| 20 \| 24.6 \| Caut et al. 2013 \| \| Western Spadefoot Toad \| *Pelobates cultripes* \| muscle \| 13C \| 1 \| 20 \| 23.3 \| Caut et al. 2013 \| \| Western Spadefoot Toad \| *Pelobates cultripes* \| muscle \| 13C \| 1 \| 20 \| 12.6 \| Caut et al. 2013 \| \| Western Spadefoot Toad \| *Pelobates cultripes* \| muscle \| 15N \| 1 \| 20 \| 16.4 \| Caut et al. 2013 \| \| Western Spadefoot Toad \| *Pelobates cultripes* \| muscle \| 15N \| 1 \| 20 \| 46.2 \| Caut et al. 2013 \| \| Western Spadefoot Toad \| *Pelobates cultripes* \| muscle \| 15N \| 1 \| 20 \| 12.0 \| Caut et al. 2013 \| \| Western Spadefoot Toad \| *Pelobates cultripes* \| muscle \| 15N \| 1 \| 20 \| 64.2 \| Caut et al. 2013 \| \| White-Footed Mouse \| *Peromyscus leucopus* \| blood \| 13C \| 25 \|  \| 16.1 \| DeMots et al. 2010 \| \| White-Footed Mouse \| *Peromyscus leucopus* \| blood \| 13C \| 25 \|  \| 14.4 \| DeMots et al. 2010 \| \| White-Footed Mouse \| *Peromyscus leucopus* \| liver \| 13C \| 25 \|  \| 5.6 \| DeMots et al. 2010 \| \| White-Footed Mouse \| *Peromyscus leucopus* \| liver \| 13C \| 25 \|  \| 3.5 \| DeMots et al. 2010 \| \| White-Footed Mouse \| *Peromyscus leucopus* \| muscle \| 13C \| 25 \|  \| 29.6 \| DeMots et al. 2010 \| \| White-Footed Mouse \| *Peromyscus leucopus* \| muscle \| 13C \| 25 \|  \| 30.1 \| DeMots et al. 2010 \| \| Whiteleg Shrimp \| *Litopenaeus vannamei* \| whole organism \| 13C \| 0.000009 \| 28.2 \| 2.5 \| Gamboa-Delgado & Le Vay 2009 \| \| Whiteleg Shrimp \| *Litopenaeus vannamei* \| whole organism \| 13C \| 0.000009 \| 28.2 \| 1.6 \| Gamboa-Delgado & Le Vay 2009 \| \| Whiteleg Shrimp \| *Litopenaeus vannamei* \| whole organism \| 13C \| 0.000009 \| 28.2 \| 1.2 \| Gamboa-Delgado & Le Vay 2009 \| \| Whiteleg Shrimp \| *Litopenaeus vannamei* \| whole organism \| 13C \| 0.000009 \| 28.2 \| 1.2 \| Gamboa-Delgado & Le Vay 2009 \| \| Whiteleg Shrimp \| *Litopenaeus vannamei* \| whole organism \| 13C \| 0.000009 \| 28.2 \| 1.4 \| Gamboa-Delgado & Le Vay 2009 \| \| Winter Flounder \| *Pseudopleuronectes americanus* \| whole organism \| 13C \| 0.00048 \| 13 \| 4.1 \| Bosley et al. 2002 \| \| Winter Flounder \| *Pseudopleuronectes americanus* \| whole organism \| 13C \| 0.00038 \| 18 \| 2.2 \| Bosley et al. 2002 \| \| Winter Flounder \| *Pseudopleuronectes americanus* \| whole organism \| 15N \| 0.00048 \| 13 \| 3.9 \| Bosley et al. 2002 \| \| Winter Flounder \| *Pseudopleuronectes americanus* \| whole organism \| 15N \| 0.00038 \| 18 \| 3.1 \| Bosley et al. 2002 \| \| Yellow Perch \| *Perca flavescens* \| muscle \| 13C \| 0.11 \| 21.5 \| 8.3 \| Weidel et al. 2011 \| \| Yellow Perch \| *Perca flavescens* \| muscle \| 13C \| 2.24 \| 21.5 \| 57.8 \| Weidel et al. 2011 \| \| Yellow Perch \| *Perca flavescens* \| muscle \| 13C \| 11.16 \| 21.5 \| 115.5 \| Weidel et al. 2011 \| \| Yellow-Rumped Warbler \| *Dendoica coronata* \| blood \| 13C \| 12.5 \|  \| 12.0 \| Pearson et al. 2003 \| \| Yellow-Rumped Warbler \| *Dendoica coronata* \| blood \| 15N \| 12.5 \|  \| 17.6 \| Pearson et al. 2003 \| \| Yellow-Rumped Warbler \| *Dendoica coronata* \| plasma \| 13C \| 12.5 \|  \| 0.5 \| Pearson et al. 2003 \| \| Yellow-Rumped Warbler \| *Dendoica coronata* \| plasma \| 15N \| 12.5 \|  \| 0.9 \| Pearson et al. 2003 \| \| Yellow-Rumped Warbler \| *Dendoica coronata* \| red blood cells \| 13C \| 12.5 \| 21 \| 10.9 \| Podlesak et al. 2005 \| \| Yellow-Rumped Warbler \| *Dendoica coronata* \| plasma \| 13C \| 12.5 \| 21 \| 1.0 \| Podlesak et al. 2005 \| \| Yellow-Vented Bulbul \| *Pycnonotus xanthopygos* \| blood cells \| 15N \| 36.5 \| 25 \| 20.1 \| Tsahar et al. 2008 \| \| Yellow-Vented Bulbul \| *Pycnonotus xanthopygos* \| blood cells \| 15N \| 36.5 \| 25 \| 16.3 \| Tsahar et al. 2008 \| \| Yellow-Vented Bulbul \| *Pycnonotus xanthopygos* \| blood cells \| 15N \| 36.5 \| 25 \| 15.1 \| Tsahar et al. 2008 \| \| Yellow-Vented Bulbul \| *Pycnonotus xanthopygos* \| plasma \| 15N \| 36.5 \| 25 \| 6.1 \| Tsahar et al. 2008 \| \| Yellow-Vented Bulbul \| *Pycnonotus xanthopygos* \| plasma \| 15N \| 36.5 \| 25 \| 3.8 \| Tsahar et al. 2008 \| \| Yellow-Vented Bulbul \| *Pycnonotus xanthopygos* \| plasma \| 15N \| 36.5 \| 25 \| 3.4 \| Tsahar et al. 2008 \| \| Zebra Danio \| *Danio rerio* \| muscle \| 13C \| 0.28 \| 28.5 \| 53.0 \| Tarboush et al. 2006 \| \| Zebra Danio \| *Danio rerio* \| muscle \| 15N \| 0.28 \| 28.5 \| 147.0 \| Tarboush et al. 2006 \| \| Zebra Danio \| *Danio rerio* \| muscle \| 34S \| 0.28 \| 28.5 \| 58.0 \| Tarboush et al. 2006 \| \| Zebra Finch \| *Taeniopygia guttata* \| red blood cells \| 13C \| 14.95 \| 32 \| 13.4 \| Bauchinger et al. 2010 \| \| Zebra Finch \| *Taeniopygia guttata* \| red blood cells \| 13C \| 14.35 \| 32 \| 11.6 \| Bauchinger et al. 2010 \| \| Zebra Finch \| *Taeniopygia guttata* \| red blood cells \| 13C \| 14.97 \| 15 \| 11.0 \| Bauchinger et al. 2010 \| \| Zebra Finch \| *Taeniopygia guttata* \| red blood cells \| 13C \| 16 \| 31 \| 13.4 \| Bauchinger & McWilliams 2009 \| \| Zebra Finch \| *Taeniopygia guttata* \| brain \| 13C \| 14.95 \| 32 \| 45.5 \| Bauchinger et al. 2010 \| \| Zebra Finch \| *Taeniopygia guttata* \| brain \| 13C \| 14.35 \| 32 \| 18.0 \| Bauchinger et al. 2010 \| \| Zebra Finch \| *Taeniopygia guttata* \| brain \| 13C \| 14.97 \| 15 \| 13.3 \| Bauchinger et al. 2010 \| \| Zebra Finch \| *Taeniopygia guttata* \| brain \| 13C \| 16 \| 31 \| 12.8 \| Bauchinger & McWilliams 2009 \| \| Zebra Finch \| *Taeniopygia guttata* \| gizzard \| 13C \| 14.95 \| 32 \| 6.9 \| Bauchinger et al. 2010 \| \| Zebra Finch \| *Taeniopygia guttata* \| gizzard \| 13C \| 14.35 \| 32 \| 7.1 \| Bauchinger et al. 2010 \| \| Zebra Finch \| *Taeniopygia guttata* \| gizzard \| 13C \| 14.97 \| 15 \| 5.0 \| Bauchinger et al. 2010 \| \| Zebra Finch \| *Taeniopygia guttata* \| gizzard \| 13C \| 16 \| 31 \| 7.0 \| Bauchinger & McWilliams 2009 \| \| Zebra Finch \| *Taeniopygia guttata* \| intestine \| 13C \| 14.95 \| 32 \| 5.6 \| Bauchinger et al. 2010 \| \| Zebra Finch \| *Taeniopygia guttata* \| intestine \| 13C \| 14.35 \| 32 \| 4.5 \| Bauchinger et al. 2010 \| \| Zebra Finch \| *Taeniopygia guttata* \| intestine \| 13C \| 14.97 \| 15 \| 4.2 \| Bauchinger et al. 2010 \| \| Zebra Finch \| *Taeniopygia guttata* \| intestine \| 13C \| 16 \| 31 \| 5.6 \| Bauchinger & McWilliams 2009 \| \| Zebra Finch \| *Taeniopygia guttata* \| proventriculus \| 13C \| 14.95 \| 32 \| 9.2 \| Bauchinger et al. 2010 \| \| Zebra Finch \| *Taeniopygia guttata* \| proventriculus \| 13C \| 14.35 \| 32 \| 11.8 \| Bauchinger et al. 2010 \| \| Zebra Finch \| *Taeniopygia guttata* \| proventriculus \| 13C \| 14.97 \| 15 \| 7.0 \| Bauchinger et al. 2010 \| \| Zebra Finch \| *Taeniopygia guttata* \| proventriculus \| 13C \| 16 \| 31 \| 9.3 \| Bauchinger & McWilliams 2009 \| \| Zebra Finch \| *Taeniopygia guttata* \| heart \| 13C \| 14.95 \| 32 \| 11.8 \| Bauchinger et al. 2010 \| \| Zebra Finch \| *Taeniopygia guttata* \| heart \| 13C \| 14.35 \| 32 \| 12.6 \| Bauchinger et al. 2010 \| \| Zebra Finch \| *Taeniopygia guttata* \| heart \| 13C \| 14.97 \| 15 \| 10.4 \| Bauchinger et al. 2010 \| \| Zebra Finch \| *Taeniopygia guttata* \| heart \| 13C \| 16 \| 31 \| 12.0 \| Bauchinger & McWilliams 2009 \| \| Zebra Finch \| *Taeniopygia guttata* \| kidney \| 13C \| 14.95 \| 32 \| 8.0 \| Bauchinger et al. 2010 \| \| Zebra Finch \| *Taeniopygia guttata* \| kidney \| 13C \| 14.35 \| 32 \| 6.7 \| Bauchinger et al. 2010 \| \| Zebra Finch \| *Taeniopygia guttata* \| kidney \| 13C \| 14.97 \| 15 \| 6.5 \| Bauchinger et al. 2010 \| \| Zebra Finch \| *Taeniopygia guttata* \| kidney \| 13C \| 16 \| 31 \| 8.0 \| Bauchinger & McWilliams 2009 \| \| Zebra Finch \| *Taeniopygia guttata* \| liver \| 13C \| 14.95 \| 32 \| 8.3 \| Bauchinger et al. 2010 \| \| Zebra Finch \| *Taeniopygia guttata* \| liver \| 13C \| 14.35 \| 32 \| 8.5 \| Bauchinger et al. 2010 \| \| Zebra Finch \| *Taeniopygia guttata* \| liver \| 13C \| 14.97 \| 15 \| 6.7 \| Bauchinger et al. 2010 \| \| Zebra Finch \| *Taeniopygia guttata* \| liver \| 13C \| 16 \| 31 \| 8.3 \| Bauchinger & McWilliams 2009 \| \| Zebra Finch \| *Taeniopygia guttata* \| pancreas \| 13C \| 14.95 \| 32 \| 7.4 \| Bauchinger et al. 2010 \| \| Zebra Finch \| *Taeniopygia guttata* \| pancreas \| 13C \| 14.35 \| 32 \| 7.5 \| Bauchinger et al. 2010 \| \| Zebra Finch \| *Taeniopygia guttata* \| pancreas \| 13C \| 14.97 \| 15 \| 5.7 \| Bauchinger et al. 2010 \| \| Zebra Finch \| *Taeniopygia guttata* \| pancreas \| 13C \| 16 \| 31 \| 8.3 \| Bauchinger & McWilliams 2009 \| \| Zebra Finch \| *Taeniopygia guttata* \| flight muscle \| 13C \| 14.95 \| 32 \| 14.6 \| Bauchinger et al. 2010 \| \| Zebra Finch \| *Taeniopygia guttata* \| flight muscle \| 13C \| 14.35 \| 32 \| 16.8 \| Bauchinger et al. 2010 \| \| Zebra Finch \| *Taeniopygia guttata* \| flight muscle \| 13C \| 14.97 \| 15 \| 13.0 \| Bauchinger et al. 2010 \| \| Zebra Finch \| *Taeniopygia guttata* \| flight muscle \| 13C \| 16 \| 31 \| 14.5 \| Bauchinger & McWilliams 2009 \| \| Zebra Finch \| *Taeniopygia guttata* \| leg muscle \| 13C \| 14.95 \| 32 \| 18.0 \| Bauchinger et al. 2010 \| \| Zebra Finch \| *Taeniopygia guttata* \| leg muscle \| 13C \| 14.35 \| 32 \| 20.4 \| Bauchinger et al. 2010 \| \| Zebra Finch \| *Taeniopygia guttata* \| leg muscle \| 13C \| 14.97 \| 15 \| 18.6 \| Bauchinger et al. 2010 \| \| Zebra Finch \| *Taeniopygia guttata* \| leg muscle \| 13C \| 16 \| 31 \| 18.1 \| Bauchinger & McWilliams 2009 \| |  |  |  |  |  |  |  |  |  |
| --- | --- | --- | --- | --- | --- | --- | --- | --- | --- | --- | --- | --- | --- | --- | --- | --- | --- | --- | --- | --- | --- | --- | --- | --- | --- | --- | --- | --- | --- | --- | --- | --- | --- | --- | --- | --- | --- | --- | --- | --- | --- | --- | --- | --- | --- | --- | --- | --- | --- | --- | --- | --- | --- | --- | --- | --- | --- | --- | --- | --- | --- | --- | --- | --- | --- | --- | --- | --- | --- | --- | --- | --- | --- | --- | --- | --- | --- | --- | --- | --- | --- | --- | --- | --- | --- | --- | --- | --- | --- | --- | --- | --- | --- | --- | --- | --- | --- | --- | --- | --- | --- | --- | --- | --- | --- | --- | --- | --- | --- | --- | --- | --- | --- | --- | --- | --- | --- | --- | --- | --- | --- | --- | --- | --- | --- | --- | --- | --- | --- | --- | --- | --- | --- | --- | --- | --- | --- | --- | --- | --- | --- | --- | --- | --- | --- | --- | --- | --- | --- | --- | --- | --- | --- | --- | --- | --- | --- | --- | --- | --- | --- | --- | --- | --- | --- | --- | --- | --- | --- | --- | --- | --- | --- | --- | --- | --- | --- | --- | --- | --- | --- | --- | --- | --- | --- | --- | --- | --- | --- | --- | --- | --- | --- | --- | --- | --- | --- | --- | --- | --- | --- | --- | --- | --- | --- | --- | --- | --- | --- | --- | --- | --- | --- | --- | --- | --- | --- | --- | --- | --- | --- | --- | --- | --- | --- | --- | --- | --- | --- | --- | --- | --- | --- | --- | --- | --- | --- | --- | --- | --- | --- | --- | --- | --- | --- | --- | --- | --- | --- | --- | --- | --- | --- | --- | --- | --- | --- | --- | --- | --- | --- | --- | --- | --- | --- | --- | --- | --- | --- | --- | --- | --- | --- | --- | --- | --- | --- | --- | --- | --- | --- | --- | --- | --- | --- | --- | --- | --- | --- | --- | --- | --- | --- | --- | --- | --- | --- | --- | --- | --- | --- | --- | --- | --- | --- | --- | --- | --- | --- | --- | --- | --- | --- | --- | --- | --- | --- | --- | --- | --- | --- | --- | --- | --- | --- | --- | --- | --- | --- | --- | --- | --- | --- | --- | --- | --- | --- | --- | --- | --- | --- | --- | --- | --- | --- | --- | --- | --- | --- | --- | --- | --- | --- | --- | --- | --- | --- | --- | --- | --- | --- | --- | --- | --- | --- | --- | --- | --- | --- | --- | --- | --- | --- | --- | --- | --- | --- | --- | --- | --- | --- | --- | --- | --- | --- | --- | --- | --- | --- | --- | --- | --- | --- | --- | --- | --- | --- | --- | --- | --- | --- | --- | --- | --- | --- | --- | --- | --- | --- | --- | --- | --- | --- | --- | --- | --- | --- | --- | --- | --- | --- | --- | --- | --- | --- | --- | --- | --- | --- | --- | --- | --- | --- | --- | --- | --- | --- | --- | --- | --- | --- | --- | --- | --- | --- | --- | --- | --- | --- | --- | --- | --- | --- | --- | --- | --- | --- | --- | --- | --- | --- | --- | --- | --- | --- | --- | --- | --- | --- | --- | --- | --- | --- | --- | --- | --- | --- | --- | --- | --- | --- | --- | --- | --- | --- | --- | --- | --- | --- | --- | --- | --- | --- | --- | --- | --- | --- | --- | --- | --- | --- | --- | --- | --- | --- | --- | --- | --- | --- | --- | --- | --- | --- | --- | --- | --- | --- | --- | --- | --- | --- | --- | --- | --- | --- | --- | --- | --- | --- | --- | --- | --- | --- | --- | --- | --- | --- | --- | --- | --- | --- | --- | --- | --- | --- | --- | --- | --- | --- | --- | --- | --- | --- | --- | --- | --- | --- | --- | --- | --- | --- | --- | --- | --- | --- | --- | --- | --- | --- | --- | --- | --- | --- | --- | --- | --- | --- | --- | --- | --- | --- | --- | --- | --- | --- | --- | --- | --- | --- | --- | --- | --- | --- | --- | --- | --- | --- | --- | --- | --- | --- | --- | --- | --- | --- | --- | --- | --- | --- | --- | --- | --- | --- | --- | --- | --- | --- | --- | --- | --- | --- | --- | --- | --- | --- | --- | --- | --- | --- | --- | --- | --- | --- | --- | --- | --- | --- | --- | --- | --- | --- | --- | --- | --- | --- | --- | --- | --- | --- | --- | --- | --- | --- | --- | --- | --- | --- | --- | --- | --- | --- | --- | --- | --- | --- | --- | --- | --- | --- | --- | --- | --- | --- | --- | --- | --- | --- | --- | --- | --- | --- | --- | --- | --- | --- | --- | --- | --- | --- | --- | --- | --- | --- | --- | --- | --- | --- | --- | --- | --- | --- | --- | --- | --- | --- | --- | --- | --- | --- | --- | --- | --- | --- | --- | --- | --- | --- | --- | --- | --- | --- | --- | --- | --- | --- | --- | --- | --- | --- | --- | --- | --- | --- | --- | --- | --- | --- | --- | --- | --- | --- | --- | --- | --- | --- | --- | --- | --- | --- | --- | --- | --- | --- | --- | --- | --- | --- | --- | --- | --- | --- | --- | --- | --- | --- | --- | --- | --- | --- | --- | --- | --- | --- | --- | --- | --- | --- | --- | --- | --- | --- | --- | --- | --- | --- | --- | --- | --- | --- | --- | --- | --- | --- | --- | --- | --- | --- | --- | --- | --- | --- | --- | --- | --- | --- | --- | --- | --- | --- | --- | --- | --- | --- | --- | --- | --- | --- | --- | --- | --- | --- | --- | --- | --- | --- | --- | --- | --- | --- | --- | --- | --- | --- | --- | --- | --- | --- | --- | --- | --- | --- | --- | --- | --- | --- | --- | --- | --- | --- | --- | --- | --- | --- | --- | --- | --- | --- | --- | --- | --- | --- | --- | --- | --- | --- | --- | --- | --- | --- | --- | --- | --- | --- | --- | --- | --- | --- | --- | --- | --- | --- | --- | --- | --- | --- | --- | --- | --- | --- | --- | --- | --- | --- | --- | --- | --- | --- | --- | --- | --- | --- | --- | --- | --- | --- | --- | --- | --- | --- | --- | --- | --- | --- | --- | --- | --- | --- | --- | --- | --- | --- | --- | --- | --- | --- | --- | --- | --- | --- | --- | --- | --- | --- | --- | --- | --- | --- | --- | --- | --- | --- | --- | --- | --- | --- | --- | --- | --- | --- | --- | --- | --- | --- | --- | --- | --- | --- | --- | --- | --- | --- | --- | --- | --- | --- | --- | --- | --- | --- | --- | --- | --- | --- | --- | --- | --- | --- | --- | --- | --- | --- | --- | --- | --- | --- | --- | --- | --- | --- | --- | --- | --- | --- | --- | --- | --- | --- | --- | --- | --- | --- | --- | --- | --- | --- | --- | --- | --- | --- | --- | --- | --- | --- | --- | --- | --- | --- | --- | --- | --- | --- | --- | --- | --- | --- | --- | --- | --- | --- | --- | --- | --- | --- | --- | --- | --- | --- | --- | --- | --- | --- | --- | --- | --- | --- | --- | --- | --- | --- | --- | --- | --- | --- | --- | --- | --- | --- | --- | --- | --- | --- | --- | --- | --- | --- | --- | --- | --- | --- | --- | --- | --- | --- | --- | --- | --- | --- | --- | --- | --- | --- | --- | --- | --- | --- | --- | --- | --- | --- | --- | --- | --- | --- | --- | --- | --- | --- | --- | --- | --- | --- | --- | --- | --- | --- | --- | --- | --- | --- | --- | --- | --- | --- | --- | --- | --- | --- | --- | --- | --- | --- | --- | --- | --- | --- | --- | --- | --- | --- | --- | --- | --- | --- | --- | --- | --- | --- | --- | --- | --- | --- | --- | --- | --- | --- | --- | --- | --- | --- | --- | --- | --- | --- | --- | --- | --- | --- | --- | --- | --- | --- | --- | --- | --- | --- | --- | --- | --- | --- | --- | --- | --- | --- | --- | --- | --- | --- | --- | --- | --- | --- | --- | --- | --- | --- | --- | --- | --- | --- | --- | --- | --- | --- | --- | --- | --- | --- | --- | --- | --- | --- | --- | --- | --- | --- | --- | --- | --- | --- | --- | --- | --- | --- | --- | --- | --- | --- | --- | --- | --- | --- | --- | --- | --- | --- | --- | --- | --- | --- | --- | --- | --- | --- | --- | --- | --- | --- | --- | --- | --- | --- | --- | --- | --- | --- | --- | --- | --- | --- | --- | --- | --- | --- | --- | --- | --- | --- | --- | --- | --- | --- | --- | --- | --- | --- | --- | --- | --- | --- | --- | --- | --- | --- | --- | --- | --- | --- | --- | --- | --- | --- | --- | --- | --- | --- | --- | --- | --- | --- | --- | --- | --- | --- | --- | --- | --- | --- | --- | --- | --- | --- | --- | --- | --- | --- | --- | --- | --- | --- | --- | --- | --- | --- | --- | --- | --- | --- | --- | --- | --- | --- | --- | --- | --- | --- | --- | --- | --- | --- | --- | --- | --- | --- | --- | --- | --- | --- | --- | --- | --- | --- | --- | --- | --- | --- | --- | --- | --- | --- | --- | --- | --- | --- | --- | --- | --- | --- | --- | --- | --- | --- | --- | --- | --- | --- | --- | --- | --- | --- | --- | --- | --- | --- | --- | --- | --- | --- | --- | --- | --- | --- | --- | --- | --- | --- | --- | --- | --- | --- | --- | --- | --- | --- | --- | --- | --- | --- | --- | --- | --- | --- | --- | --- | --- | --- | --- | --- | --- | --- | --- | --- | --- | --- | --- | --- | --- | --- | --- | --- | --- | --- | --- | --- | --- | --- | --- | --- | --- | --- | --- | --- | --- | --- | --- | --- | --- | --- | --- | --- | --- | --- | --- | --- | --- | --- | --- | --- | --- | --- | --- | --- | --- | --- | --- | --- | --- | --- | --- | --- | --- | --- | --- | --- | --- | --- | --- | --- | --- | --- | --- | --- | --- | --- | --- | --- | --- | --- | --- | --- | --- | --- | --- | --- | --- | --- | --- | --- | --- | --- | --- | --- | --- | --- | --- | --- | --- | --- | --- | --- | --- | --- | --- | --- | --- | --- | --- | --- | --- | --- | --- | --- | --- | --- | --- | --- | --- | --- | --- | --- | --- | --- | --- | --- | --- | --- | --- | --- | --- | --- | --- | --- | --- | --- | --- | --- | --- | --- | --- | --- | --- | --- | --- | --- | --- | --- | --- | --- | --- | --- | --- | --- | --- | --- | --- | --- | --- | --- | --- | --- | --- | --- | --- | --- | --- | --- | --- | --- | --- | --- | --- | --- | --- | --- | --- | --- | --- | --- | --- | --- | --- | --- | --- | --- | --- | --- | --- | --- | --- | --- | --- | --- | --- | --- | --- | --- | --- | --- | --- | --- | --- | --- | --- | --- | --- | --- | --- | --- | --- | --- | --- | --- | --- | --- | --- | --- | --- | --- | --- | --- | --- | --- | --- | --- | --- | --- | --- | --- | --- | --- | --- | --- | --- | --- | --- | --- | --- | --- | --- | --- | --- | --- | --- | --- | --- | --- | --- | --- | --- | --- | --- | --- | --- | --- | --- | --- | --- | --- | --- | --- | --- | --- | --- | --- | --- | --- | --- | --- | --- | --- | --- | --- | --- | --- | --- | --- | --- | --- | --- | --- | --- | --- | --- | --- | --- | --- | --- | --- | --- | --- | --- | --- | --- | --- | --- | --- | --- | --- | --- | --- | --- | --- | --- | --- | --- | --- | --- | --- | --- | --- | --- | --- | --- | --- | --- | --- | --- | --- | --- | --- | --- | --- | --- | --- | --- | --- | --- | --- | --- | --- | --- | --- | --- | --- | --- | --- | --- | --- | --- | --- | --- | --- | --- | --- | --- | --- | --- | --- | --- | --- | --- | --- | --- | --- | --- | --- | --- | --- | --- | --- | --- | --- | --- | --- | --- | --- | --- | --- | --- | --- | --- | --- | --- | --- | --- | --- | --- | --- | --- | --- | --- | --- | --- | --- | --- | --- | --- | --- | --- | --- | --- | --- | --- | --- | --- | --- | --- | --- | --- | --- | --- | --- | --- | --- | --- | --- | --- | --- | --- | --- | --- | --- | --- | --- | --- | --- | --- | --- | --- | --- | --- | --- | --- | --- | --- | --- | --- | --- | --- | --- | --- | --- | --- | --- | --- | --- | --- | --- | --- | --- | --- | --- | --- | --- | --- | --- | --- | --- | --- | --- | --- | --- | --- | --- | --- | --- | --- | --- | --- | --- | --- | --- | --- | --- | --- | --- | --- | --- | --- | --- | --- | --- | --- | --- | --- | --- | --- | --- | --- | --- | --- | --- | --- | --- | --- | --- | --- | --- | --- | --- | --- | --- | --- | --- | --- | --- | --- | --- | --- | --- | --- | --- | --- | --- | --- | --- | --- | --- | --- | --- | --- | --- | --- | --- | --- | --- | --- | --- | --- | --- | --- | --- | --- | --- | --- | --- | --- | --- | --- | --- | --- | --- | --- | --- | --- | --- | --- | --- | --- | --- | --- | --- | --- | --- | --- | --- | --- | --- | --- | --- | --- | --- | --- | --- | --- | --- | --- | --- | --- | --- | --- | --- | --- | --- | --- | --- | --- | --- | --- | --- | --- | --- | --- | --- | --- | --- | --- | --- | --- | --- | --- | --- | --- | --- | --- | --- | --- | --- | --- | --- | --- | --- | --- | --- | --- | --- | --- | --- | --- | --- | --- | --- | --- | --- | --- | --- | --- | --- | --- | --- | --- | --- | --- | --- | --- | --- | --- | --- | --- | --- | --- | --- | --- | --- | --- | --- | --- | --- | --- | --- | --- | --- | --- | --- | --- | --- | --- | --- | --- | --- | --- | --- | --- | --- | --- | --- | --- | --- | --- | --- | --- | --- | --- | --- | --- | --- | --- | --- | --- | --- | --- | --- | --- | --- | --- | --- | --- | --- | --- | --- | --- | --- | --- | --- | --- | --- | --- | --- | --- | --- | --- | --- | --- | --- | --- | --- | --- | --- | --- | --- | --- | --- | --- | --- | --- | --- | --- | --- | --- | --- | --- | --- | --- | --- | --- | --- | --- | --- | --- | --- | --- | --- | --- | --- | --- | --- | --- | --- | --- | --- | --- | --- | --- | --- | --- | --- | --- | --- | --- | --- | --- | --- | --- | --- | --- | --- | --- | --- | --- | --- | --- | --- | --- | --- | --- | --- | --- | --- | --- | --- | --- | --- | --- | --- | --- | --- | --- | --- | --- | --- | --- | --- | --- | --- | --- | --- | --- | --- | --- | --- | --- | --- | --- | --- | --- | --- | --- | --- | --- | --- | --- | --- | --- | --- | --- | --- | --- | --- | --- | --- | --- | --- | --- | --- | --- | --- | --- | --- | --- | --- | --- | --- | --- | --- | --- | --- | --- | --- | --- | --- | --- | --- | --- | --- | --- | --- | --- | --- | --- | --- | --- | --- | --- | --- | --- | --- | --- | --- | --- | --- | --- | --- | --- | --- | --- | --- | --- | --- | --- | --- | --- | --- | --- | --- | --- | --- | --- | --- | --- | --- | --- | --- | --- | --- | --- | --- | --- | --- | --- | --- | --- | --- | --- | --- | --- | --- | --- | --- | --- | --- | --- | --- | --- | --- | --- | --- | --- | --- | --- | --- | --- | --- | --- | --- | --- | --- | --- | --- | --- | --- | --- | --- | --- | --- | --- | --- | --- | --- | --- | --- | --- | --- | --- | --- | --- | --- | --- | --- | --- | --- | --- | --- | --- | --- | --- | --- | --- | --- | --- | --- | --- | --- | --- | --- | --- | --- | --- | --- | --- | --- | --- | --- | --- | --- | --- | --- | --- | --- | --- | --- | --- | --- | --- | --- | --- | --- | --- | --- | --- | --- | --- | --- | --- | --- | --- | --- | --- | --- | --- | --- | --- | --- | --- | --- | --- | --- | --- | --- | --- | --- | --- | --- | --- | --- | --- | --- | --- | --- | --- | --- | --- | --- | --- | --- | --- | --- | --- | --- | --- | --- | --- | --- | --- | --- | --- | --- | --- | --- | --- | --- | --- | --- | --- | --- | --- | --- | --- | --- | --- | --- | --- | --- | --- | --- | --- | --- | --- | --- | --- | --- | --- | --- | --- | --- | --- | --- | --- | --- | --- | --- | --- | --- | --- | --- | --- | --- | --- | --- | --- | --- | --- | --- | --- | --- | --- | --- | --- | --- | --- | --- | --- | --- | --- | --- | --- | --- | --- | --- | --- | --- | --- | --- | --- | --- | --- | --- | --- | --- | --- | --- | --- | --- | --- | --- | --- | --- | --- | --- | --- | --- | --- | --- | --- | --- | --- | --- | --- | --- | --- | --- | --- | --- | --- | --- | --- | --- | --- | --- | --- | --- | --- | --- | --- | --- | --- | --- | --- | --- | --- | --- | --- | --- | --- | --- | --- | --- | --- | --- | --- | --- | --- | --- | --- | --- | --- | --- | --- | --- | --- | --- | --- | --- | --- | --- | --- | --- | --- | --- | --- | --- | --- | --- | --- | --- | --- | --- | --- | --- | --- | --- | --- | --- | --- | --- | --- | --- | --- | --- | --- | --- | --- | --- | --- | --- | --- | --- | --- | --- | --- | --- | --- | --- | --- | --- | --- | --- | --- | --- | --- | --- | --- | --- | --- | --- | --- | --- | --- | --- | --- | --- | --- | --- | --- | --- | --- | --- | --- | --- | --- | --- | --- | --- | --- | --- | --- | --- | --- | --- | --- | --- | --- | --- | --- | --- | --- | --- | --- | --- | --- | --- | --- | --- | --- | --- | --- | --- | --- | --- | --- | --- | --- | --- | --- | --- | --- | --- | --- | --- | --- | --- | --- | --- | --- | --- | --- | --- | --- | --- | --- | --- | --- | --- | --- | --- | --- | --- | --- | --- | --- | --- | --- | --- | --- | --- | --- | --- | --- | --- | --- | --- | --- | --- | --- | --- | --- | --- | --- | --- | --- | --- | --- | --- | --- | --- | --- | --- | --- | --- | --- | --- | --- | --- | --- | --- | --- | --- | --- | --- | --- | --- | --- | --- | --- | --- | --- | --- | --- | --- | --- | --- | --- | --- | --- | --- | --- | --- | --- | --- | --- | --- | --- | --- | --- | --- | --- | --- | --- | --- | --- | --- | --- | --- | --- | --- | --- | --- | --- | --- | --- | --- | --- | --- | --- | --- | --- | --- | --- | --- | --- | --- | --- | --- | --- | --- | --- | --- | --- | --- | --- | --- | --- | --- | --- | --- | --- | --- | --- | --- | --- | --- | --- | --- | --- | --- | --- | --- | --- | --- | --- | --- | --- | --- | --- | --- | --- | --- | --- | --- | --- | --- | --- | --- | --- | --- | --- | --- | --- | --- | --- | --- | --- | --- | --- | --- | --- | --- | --- | --- | --- | --- | --- | --- | --- | --- | --- | --- | --- | --- | --- | --- | --- | --- | --- | --- | --- | --- | --- | --- | --- | --- | --- | --- | --- | --- | --- | --- | --- | --- | --- | --- | --- | --- | --- | --- | --- | --- | --- | --- | --- | --- | --- | --- | --- | --- | --- | --- | --- | --- | --- | --- | --- | --- | --- | --- | --- | --- | --- | --- | --- | --- | --- | --- | --- | --- | --- | --- | --- | --- | --- | --- | --- | --- | --- | --- | --- | --- | --- | --- | --- | --- | --- | --- | --- | --- | --- | --- | --- | --- | --- | --- | --- | --- | --- | --- | --- | --- | --- | --- | --- | --- | --- | --- | --- | --- | --- | --- | --- | --- | --- | --- | --- | --- | --- | --- | --- | --- | --- | --- | --- | --- | --- | --- | --- | --- | --- | --- | --- | --- | --- | --- | --- | --- | --- | --- | --- | --- | --- | --- | --- | --- | --- | --- | --- | --- | --- | --- | --- | --- | --- | --- | --- | --- | --- | --- | --- | --- | --- | --- | --- | --- | --- | --- | --- | --- | --- | --- | --- | --- | --- | --- | --- | --- | --- | --- | --- | --- | --- | --- | --- | --- | --- | --- | --- | --- | --- | --- | --- | --- | --- | --- | --- | --- | --- | --- | --- | --- | --- | --- | --- | --- | --- | --- | --- | --- | --- | --- | --- | --- | --- | --- | --- | --- | --- | --- | --- | --- | --- | --- | --- | --- | --- | --- | --- | --- | --- | --- | --- | --- | --- | --- | --- | --- | --- | --- | --- | --- | --- | --- | --- | --- | --- | --- | --- | --- | --- | --- | --- | --- | --- | --- | --- | --- | --- | --- | --- | --- | --- | --- | --- | --- | --- | --- | --- | --- | --- | --- | --- | --- | --- | --- | --- | --- | --- | --- | --- | --- | --- | --- | --- | --- | --- | --- | --- | --- | --- | --- | --- | --- | --- | --- | --- | --- | --- | --- | --- | --- | --- | --- | --- | --- | --- | --- | --- | --- | --- | --- | --- | --- | --- | --- | --- | --- | --- | --- | --- | --- | --- | --- | --- | --- | --- | --- | --- | --- | --- | --- | --- | --- | --- | --- | --- | --- | --- | --- | --- | --- | --- | --- | --- | --- | --- | --- | --- | --- | --- | --- | --- | --- | --- | --- | --- | --- | --- | --- | --- | --- | --- | --- | --- | --- | --- | --- | --- | --- | --- | --- | --- | --- | --- | --- | --- | --- | --- | --- | --- | --- | --- | --- | --- | --- | --- | --- | --- | --- | --- | --- | --- | --- | --- | --- | --- | --- | --- | --- | --- | --- | --- | --- | --- | --- | --- | --- | --- | --- | --- | --- | --- | --- | --- | --- | --- | --- | --- | --- | --- | --- | --- | --- | --- | --- | --- | --- | --- | --- | --- | --- | --- | --- | --- | --- | --- | --- | --- | --- | --- | --- | --- | --- | --- | --- | --- | --- | --- | --- | --- | --- | --- | --- | --- | --- | --- | --- | --- | --- | --- | --- | --- | --- | --- | --- | --- | --- | --- | --- | --- | --- | --- | --- | --- | --- | --- | --- | --- | --- | --- | --- | --- | --- | --- | --- | --- | --- | --- | --- | --- | --- | --- | --- | --- | --- | --- | --- | --- | --- | --- | --- | --- | --- | --- | --- | --- | --- | --- | --- | --- | --- | --- | --- | --- | --- | --- | --- | --- | --- | --- | --- | --- | --- | --- | --- | --- | --- | --- | --- | --- | --- | --- | --- | --- | --- | --- | --- | --- | --- | --- | --- | --- | --- | --- | --- | --- | --- | --- | --- | --- | --- | --- | --- | --- | --- | --- | --- | --- | --- | --- | --- | --- | --- | --- | --- | --- | --- | --- | --- | --- | --- | --- | --- | --- | --- | --- | --- | --- | --- | --- | --- | --- | --- | --- | --- | --- | --- | --- | --- | --- | --- | --- | --- | --- | --- | --- | --- | --- | --- | --- | --- | --- | --- | --- | --- | --- | --- | --- | --- | --- | --- | --- | --- | --- | --- | --- | --- | --- | --- | --- | --- | --- | --- | --- | --- | --- | --- | --- | --- | --- | --- | --- | --- | --- | --- | --- | --- | --- | --- | --- | --- | --- | --- | --- | --- | --- | --- | --- | --- | --- | --- | --- | --- | --- | --- | --- | --- | --- | --- | --- | --- | --- | --- | --- | --- | --- | --- | --- | --- | --- | --- | --- | --- | --- | --- | --- | --- | --- | --- | --- | --- | --- | --- | --- | --- | --- | --- | --- | --- | --- | --- | --- | --- | --- | --- | --- | --- | --- | --- | --- | --- | --- | --- | --- | --- | --- | --- | --- | --- | --- | --- | --- | --- | --- | --- | --- | --- | --- | --- | --- | --- | --- | --- | --- | --- | --- | --- | --- | --- | --- | --- | --- | --- | --- | --- | --- | --- | --- | --- | --- | --- | --- | --- | --- | --- | --- | --- | --- | --- | --- | --- | --- | --- | --- | --- | --- | --- | --- | --- | --- | --- | --- | --- | --- | --- | --- | --- | --- | --- | --- | --- | --- | --- | --- | --- | --- | --- | --- | --- | --- | --- | --- | --- | --- | --- | --- | --- | --- | --- | --- | --- | --- | --- | --- | --- | --- | --- | --- | --- | --- | --- | --- | --- | --- | --- | --- | --- | --- | --- | --- | --- | --- | --- | --- | --- | --- | --- | --- | --- | --- | --- | --- | --- | --- | --- | --- | --- | --- | --- | --- | --- | --- | --- | --- | --- | --- | --- | --- | --- | --- | --- | --- | --- | --- | --- | --- | --- | --- | --- | --- | --- | --- | --- | --- | --- | --- | --- | --- | --- | --- | --- | --- | --- | --- | --- | --- | --- | --- | --- | --- | --- | --- | --- | --- | --- | --- | --- | --- | --- | --- | --- | --- | --- | --- | --- | --- | --- | --- | --- | --- | --- | --- | --- | --- | --- | --- | --- | --- | --- | --- | --- | --- | --- | --- | --- | --- | --- | --- | --- | --- | --- | --- | --- | --- | --- | --- | --- | --- | --- | --- | --- | --- | --- | --- | --- | --- | --- | --- | --- | --- | --- | --- | --- | --- | --- | --- | --- | --- | --- | --- | --- | --- | --- | --- | --- | --- | --- | --- | --- | --- | --- | --- | --- | --- | --- | --- | --- | --- | --- | --- | --- | --- | --- | --- | --- | --- | --- | --- | --- | --- | --- | --- | --- | --- | --- | --- | --- | --- | --- | --- | --- | --- | --- | --- | --- | --- | --- | --- | --- | --- | --- | --- | --- | --- | --- | --- | --- | --- | --- | --- | --- | --- | --- | --- | --- | --- | --- | --- | --- | --- | --- | --- | --- | --- | --- | --- | --- | --- | --- | --- | --- | --- | --- | --- | --- | --- | --- | --- | --- | --- | --- | --- | --- | --- | --- | --- | --- | --- | --- | --- | --- | --- | --- | --- | --- | --- | --- | --- | --- | --- | --- | --- | --- | --- | --- | --- | --- | --- | --- | --- | --- | --- | --- | --- | --- | --- | --- | --- | --- | --- | --- | --- | --- | --- | --- | --- | --- | --- | --- | --- | --- | --- | --- | --- | --- | --- | --- | --- | --- | --- | --- | --- | --- | --- | --- | --- | --- | --- | --- | --- | --- |
|  |  |  |  |  |  |  |  |  |  |

**References in Appendix**

Ankjaerø T, Christensen JT, Grønkjær P (2012) Tissue-specific turnover rates and trophic enrichment of stable N and C isotopes in juvenile Atlantic cod *Gadus morhua* fed three different diets. Mar Ecol Prog Ser 461:197-209

Arneson LS, MacAvoy SE, Bassett E (2006) Metabolic protein replacement drives tissue turnover in adult mice. Can J Zool 84:992-1002

Bahar B, Moloney AP, Monahan FJ, Harrison SM, Zazzo A, Scrimgeour CM, Begley IS, Schmidt O (2009) Turnover of carbon, nitrogen, and sulfur in bovine longissimus dorsi and psoas major muscles: Implications for isotopic authentication of meat. J Anim Sci 87:905-913

Barquete V, Strauss V, Ryan PG (2012) Stable isotope turnover in blood and claws: a case study in captive African Penguins. J Exp Mar Biol Ecol 448:121-127

Bauchinger U, McWilliams SR (2009) Carbon turnover in tissues of a passerine bird: Allometry, isotopic clocks, and phenotypic plasticity in organ size. Physiol Biochem Zool 82:787-797

Bauchinger U, Keil J, McKinney RA, Starck JM, McWilliams SR (2010) Exposure to cold but not exercise increases carbon turnover rates in specific tissues of a passerine. J Exp Biol 213:526-534

Bearhop S, Waldron S, Votier SC, Furness RW (2002) Factors that influence assimilation rates and fractionation of nitrogen and carbon stable isotopes in avian blood and feathers. Physiol Biochem Zool 75:451-458

Bosley KL, Witting DA, Chambers RC, Wainwright SC (2002) Estimating turnover rates of carbon and nitrogen in recently metamorphosed winter flounder *Pseudopleuronectes americanus* with stable isotopes. Mar Ecol Prog Ser 236:233-240

Braun A, Schneider S, Auerswald K, Bellof G, Schnyder H (2013) Dietary protein content affects isotopic carbon and nitrogen turnover Rapid Comm Mass Spectrom 27: 2676-2684

Buchheister A, Latour RJ (2010) Turnover and fractionation of carbon and nitrogen stable isotopes in tissues of a migratory coastal predator, summer flounder (*Paralichthys dentatus*). Can J Fish Aquat Sci 67:445-461

Carleton SA, Martínez del Río C (2005) The effect of cold-induced increased metabolic rate on the rate of (13)C and (15)N incorporation in house sparrows (Passer domesticus). Oecologia 144:226-232

Carleton SA, Kelly L, Anderson-Sprecher R, Martínez del Río C (2008) Should we use one-, or multi-compartment models to describe 13C incorporation into animal tissues? Rapid Commun Mass Spectrom 22:3008-3014

Carleton SA, Martínez del Río C (2010) Growth and catabolism in isotopic incorporation: a new formulation and experimental data. Funct Ecol 24:805-812

Caut S, Angulo E, Díaz-Paniagua C, Gomez-Mestre I (2013) Plastic changes in tadpole trophic ecology revealed by stable isotope analysis. Oecologia 173:95-105

Church MR, Ebersole JL, Rensmeyer KM, Couture RB, Barrows FT, Noakes DLG (2009) Mucus: A new tissue fractionation for rapid determination of fish diet switching using stable isotope analysis. Can J Fish Aquat Sci 66:1-5

Cruz VC, Ducatti C, Pezzato AC, Pinheiro DF, Sartori JR, Goncalves JC, Carrijo AS (2005) Influence of diet on assimilation and turnover of C-13 in the tissues of broiler chickens. Br Poult Sci 46:382-389

DeMots RL, Novak JM, Gaines KF, Gregor AJ, Romanek CS, Soluk DA (2010) Tissue-diet discrimination factors and turnover of stable carbon and nitrogen isotopes in white-footed mice (*Peromyscus leucopus*). Can J Zool 88:961-967

Dubois S, Jean-Louis B, Bertrand B, Lefebvre S (2007) Isotope trophic-step fractionation of suspension-feeding species: Implications for food partitioning in coastal ecosystems. J Exp Mar Biol Ecol 351:212-128

Fisk AT, Sash K, Maerz J, Palmer W, Carroll JP, MacNeil MA (2009) Metabolic turnover rates of carbon and nitrogen stable isotopes in captive juvenile snakes. Rapid Comm Mass Spectrom 23:319-326

Fry B, Arnold C (1982) Rapid C-13/C-12 turnover during growth of brown shrimp (*Penaeus aztecus*). Oecologia 54:200-204

Furuya VRB, Hayashi C, Furuya WM, Sakaguti ES (2002) Replacement rates of carbon stable isotope (13-C) in muscle tissue of pintado, *Pseudoplatystoma corruscans* (Agassiz, 1829). Zootec Trop 20:461-472

Gamboa-Delgado J, Canavate JP, Zerolo R, Le Vay L (2008) Natural carbon stable isotope ratios as indicators of the relative contribution of live and inert diets to growth in larval Senegalese sole (*Solea senegalensis*). Aquaculture 280:190-197

Gamboa-Delgado J, Le Vay L (2009) Natural stable isotopes as indicators of the relative contribution of soy protein and fish meal to tissue growth in Pacific white shrimp (*Litopenaeus vannamei*) fed compound diets. Aquaculture 291:115-123

German DP, Miles RD (2010) Stable carbon and nitrogen incorporation in blood and fin tissue of the catfish *Pterygoplichthys disjunctivus* (Siluriformes, Loricariidae). Environ Biol Fish 89:117-133

Guelinckx J, Maes J, Van Den Driessche P, Geysen B, Deharis F, Ollevier F (2007) Changes in delta C-13 and delta N-15 in different tissues of juvenile sand goby *Pomatoschistus minutus*: a laboratory diet-switch experiment. Mar Ecol Prog Ser 241:205-215

Harrison SM, Schmidt O, Moloney AP, Kelly SD, Rossmann A, Schellenberg A, Camin F, Perini M, Hoogewerff J, Monahan FJ (2011) Tissue turnover in ovine muscles and lipids as recorded by multiple (H, C, O, S) stable isotope ratios. Food Chem 124:291-297

Hakvoort SGH, Schmidt O (2002) N-15 isotope labeling of slugs (Gastropoda : Pulmonata). Ann Appl Biol 141:275-281

Heady WN, Moore JW (2013) Tissue turnover and stable isotope clocks to quantify resource shifts in anadromous rainbow trout. Oecologia 172:21-34

Herzka SZ, Holt GJ (2000) Changes in isotopic composition of red drum (*Sciaenops ocellatus*) larvae in response to dietary shifts: potential application to settlement studies. Can J Fish Aquat Sci 57:137-147

Herzka SZ, Holt SA, Holt GJ (2001) Documenting the settlement history of individual fish larvae using stable isotope ratios: model development and validation. J Exp Mar Biol Ecol 265:49-74

Hesslein RH, Hallard KA, Ramlal P (1993) Replacement of sulfur, carbon, and nitrogen in tissue of growing broad whitefish (*Coregonus nasus*) in response to a change in diet traced by delta S-34, delta C-13, and delta N-15. Can J Fish Aquat Sci 50:2071-2076

Hilderbrand GV, Farley SD, Robbins CT, Hanley TA, Titus K, Servheen C (1996) Use of stable isotopes to determine diets of living and extinct bears. Can J Zool-Rev Can Zool 74:2080-2088

Hobson KA, Clark RG (1993) Turnover of C-13 in Cellular and Plasma Fractions of Blood - Implications for Nondestructive Sampling in Avian Dietary Studies. Auk 110:638-641

Hobson KA, Bairlein F (2003) Isotopic fractionation and turnover in captive garden warblers (*Sylvia borin*): Implications for delineating dietary and migratory associations in wild passerines. Can J Zool-Rev Can Zool 81:1630-1635

Hobson KA, Clark RG (1992) Assessing avian diets using stable isotopes. 1. Turnover of C-13 in tissues. Condor 94:181-188

Jardine TD, Kidd KA, Polhemus JT, Cunjak RA (2008) An elemental and stable isotope assessment of water strider feeding ecology and lipid dynamics: Synthesis of laboratory and field studies. Freshw Biol 53:2192-2205

Jardine TD, MacLatchy DL, Fairchild WL, Cunjak RA, Brown SB (2004) Rapid carbon turnover during growth of Atlantic salmon (S*almo salar*) smolts in sea water, and evidence for reduced food consumption by growth-stunts. Hydrobiologia 527:63-75

Kaufman MR, Gradinger RR, Bluhm BA, O’Brien DM (2008) Using stable isotopes to assess carbon and nitrogen turnover in the Arctic sympagic amphipod *Onisimus litoralis*. Oecologia 158:11-22

Kim SL, Martínez del Río C, Casper D, Koch PL (2012) Isotopic incorporation rates for shark tissues from a long-term captive feeding study. J Exp Biol 215:2495-2500

Klaassen M, Thums M, Hume ID (2004) Effects of diet change on carbon and nitrogen stable-isotope ratios in blood cells and plasma of the long-nosed bandicoot (*Parameles nasuta*). Aust J Zool 52:635-647

Larsen T, Ventura M, O’Brien DM, Magid J, Lomstein BA, Larsen J (2011) Contrasting effects of nitrogen limitation and amino acid imbalance on carbon and nitrogen turnover in three species of Collembola. Soil Biol Biochem 43:749-759

Lecomte N, Ahlstrøm O, Ehrich D, Fuglei E, Ims RA, Yoccoz NG (2011) Intrapopulation variability shaping isotope discrimination and turnover: Experimental evidence in Arctic foxes. PLoS ONE 6:e21357

Logan JM, Lutcavage ME (2010) Stable isotope dynamics in elasmobranch fishes. Hydrobiologia 644:231-244

MacAvoy SE, Macko SA, Garman SC (2001) Isotopic turnover in aquatic predators: Quantifying the exploitation of migratory prey. Can J Fish Aquat Sci 58:923-932

MacAvoy SE, Macko SA, Arneson LS (2005) Growth versus metabolic tissue replacement in mouse tissues determined by stable carbon and nitrogen isotope analysis. Can J Zool-Rev Can Zool 83:631-641

MacAvoy SE, Arneson LS, Bassett E (2006) Correlation of metabolism with tissue carbon and nitrogen turnover rate in small mammals. Oecologia 150:190-201

MacAvoy SE, Lazaroff S, Kraeer K, Arneson LS (2012) Sex and strain differences in isotope turnover rates and metabolism in house mice (*Mus musculus*). Can J Zool 90:984-990

MacNeil MA, Drouillard KG, Fisk AT (2006) Variable uptake and elimination of stable nitrogen isotopes between tissues in fish. Can J Fish Aquat Sci 63:345-353

Madeira F, Di Lascio A, Carlino P, Constantini ML, Pons X (2013) Change in carbon stable isotope ratios of the predatory bug *Orius majusculus* after dietary shifts. Entomol Exp Appl 148:287-296

Madigan DJ, Litvin SY, Popp BN, Carlisle AB, Farwell CJ, Block BA (2012) Tissue turnover rates and isotopic trophic discrimination in the endothermic teleost, Pacific Bluefin Tuna (*Thunnus orientalis*). PLoS ONE 7:e49220

Malpica-Cruz L, Herzka SZ, Sosa-Nishizaki O, Lazo JP (2012) Tissue-specific isotope trophic discrimination factors and turnover rates in a marine elasmobranch: empirical and modeling results. Can J Fish Aquat Sci 69:551-64

Maruyama A, Yamada Y, Rusuwa B, Yuma M (2001) Change in stable nitrogen isotope ratio in the muscle tissue of a migratory goby, *Rhinogobius sp.*, in a natural setting. Can J Fish Aquat Sci 58:2125-2128

McIntyre PB, Flecker AS (2006) Rapid turnover of tissue nitrogen of primary consumers in tropical freshwaters. Oecologia 148:12-21

Miller TW (2000) Tissue-specific response of δ15N in adult Pacific herring (*Clupea pallasi*) following an isotopic shift in diet. Environ Biol Fish 76:177-189

Miller JF, Millar JS, Longstaffe FJ (2008) Carbon- and nitrogen-isotope tissue-diet discrimination and turnover rates in deer mice, *Peromyscus maniculatus*. Can J Zool-Rev Can Zool 86:685-691

Miron MLL, Herrera MLG, Ramirez PN, Hobson KA (2006) Effect of diet quality on carbon and nitrogen turnover and isotopic discrimination in blood of a New World nectarivorous bat. J Exp Biol 209:541-548

Murray IW, Wolf BO (2012) Tissue carbon incorporation rates and diet-to-tissue discrimination in ectotherms: Tortoises are really slow. Physiol Biochem Zool 85:96-105

Murray IW, Wolf BO (2013) Diet and growth influence carbon incorporation rates and discrimination factors (Δ13C) in desert box turtles *Terrapene ornata luteola*. Herpetol Conserv Biol 8:149-162

Nelson J, Chanton J, Coleman F, Koenig C (2011) Patterns of stable carbon isotope turnover in gag, *Mycteroperca microlepis*, an economically important marine piscivore determined with a non-lethal surgical biopsy procedure. Environ Biol Fish 90:243-252

Ogden LJE, Hobson KA, Lank DB (2004) Blood isotopic (delta C-13 and delta C-15) turnover and diet-tissue fractionation factors in captive dunlin (*Calidris alpina pacifica*). Auk 121:170-177

Olive PJW, Pinnegar JK, Polunin PVC, Richards G, Welch R (2003) Isotope trophic-step fractionation: A dynamic equilibrium model. J Anim Ecol 72:608-617

Overmyer JP, MacNeil MA, Fisk AT (2008) Fractionation and metabolic turnover of carbon and nitrogen stable isotopes in black fly larvae. Rapid Comm Mass Spectrom 22:694-700

Pearson SF, Levey DJ, Greenberg CH, Martínez del Río C (2003) Effects of elemental composition on the incorporation of dietary nitrogen and carbon isotopic signatures in an omnivorous songbird. Oecologia 135:516-523

Podlesak DW, McWilliams SR, Hatch KA (2005) Stable isotopes in breath, blood, feces and feather can indicate intra-individual changes in the diet of migratory songbirds. Oecologia 142:501-510

Reich KJ, Bjorndal KA, Martínez del Río C (2008) Effects of growth and tissue type on the kinetics of 13C and 15N incorporation in a rapidly growing ectotherm. Oecologia 155:651-663

Rosenblatt AE, Heithaus MR (2012) Slow isotope turnover rates and low discrimination values in the American Alligator: Implications for interpretation of ectotherm stable isotope data. Physiol Biochem Zool 86:137-148

Sakano H, Fujiwara E, Nohara S, Ueda H (2005) Estimation of nitrogen stable isotope turnover rate of *Oncorhynchus nerka*. Environ Biol Fish 72:13-18

Schmidt O, Scrimgeour CM, Curry JP (1999) Carbon and nitrogen stable isotope ratios in body tissue and mucus of feeding and fasting earthworms (*Lumbricus festivus*). Oecologia 118:9-15

Seminoff JA, Bjorndal KA, Bolten AB (2007) Stable carbon and nitrogen isotope discrimination and turnover in pond sliders *Trachemys scripta*: Insights for trophic study of freshwater turtles. Copeia 2007:534-542

Sponheimer M, Robinson TF, Cerling TE, Tegland L, Roeder BL, Ayliffe L, Dearing MD, Ehleringer JD (2006) Turnover of stable carbon isotopes in the muscle, liver, and breath CO2 of alpacas (*Lama pacos*). Rapid Comm Mass Spectrom 20:1395-1399

Sun Z-L, Gao Q-F, Dong S-L, Shin PKS, Wang F (2012) Estimates of carbon turnover rates in the sea cucumber *Apostichopus japonicus* (Selenka) using stable isotope analysis: the role of metabolism and growth. Mar Ecol Prog Ser 457:101-112

Suring E, Wing SR (2009) Isotopic turnover rate and fractionation in multiple tissues of red rock lobster (*Jasus edwardsii*) and blue cod (*Parapercis colias*): Consequences for ecological studies. J Exp Mar Biol Ecol 370:56-63

Suzuki KW, Kasai A, Nakayama K, Tanaka M (2005) Differential isotopic enrichment and half-life among tissues in Japanese temperate bass (*Lateolabrax japonicus*) juveniles: implications for analyzing migration. Can J Fish Aquat Sci 62:671-678

Sweeting CJ, Jennings S, Polunin NVC (2005) Variance in isotopic signatures as a descriptor of tissue turnover and degree of omnivory. Funct Ecol 19:777-784

Tarboush RA, MacAvoy SE, Macko SA, Connaughton V (2006) Contribution of catabolic tissue replacement to the turnover of stable isotopes in *Danio rerio*. Can J Zool 84:1453-1460

Tieszen LL, Boutton TW, Tesdahl KG, Slade NA (1983) Fractionation and turnover of stable carbon isotopes in animal tissues – implications for delta-C-13 analysis of diet. Oecologia 57:32-37

Tominaga O, Uno N, Seikai T (2003) Influence of diet shift from formulated feed to live mysids on the carbon and nitrogen stable isotope ratio (delta C-13 and delta N-15) in dorsal muscles of juvenile Japanese flounders, *Paralichthys olivaceus*. Aquaculture 218:265-276

Tsahar E, Wolf N, Izhaki I, Arad Z, Martínez del Río C (2008) Dietary protein influences the rate of 15N incorporation in blood cells and plasma of Yellow-Vented Bulbuls (*Pycnonotus xanthopygos*). J Exp Biol 211:459-465

Vander Zanden MJ, Hulshof M, Ridgway MS, Rasmussen JB (1998) Application of stable isotope techniques to trophic studies of age-0 smallmouth bass. Trans Amer Fish Soc 127:729-739

Voigt CC, Matt F, Michener R, Kunz TH (2003) Low turnover rates of carbon isotopes in tissues of two nectar-feeding bat species. J Exp Biol 206:1419-1427

Warne RW, Gilman CA, Wolf BO (2012) Tissue-carbon incorporation rates in lizards: Implications for ecological studies using stable isotopes in terrestrial ectotherms. Physiol Biochem Zool 83:608-617

Weidel BC, Carpenter SR, Kitchell JF, Vander Zanden MJ (2011) Rates and components of carbon turnover in fish muscle: insights from bioenergetics models and a whole-lake 13C addition. Can J Fish Aquat Sci 68:387-399

Witting DA, Chambers RC, Bosley KL, Wainright SC (2004) Experimental evaluation of ontogenetic diet transitions in summer flounder (*Paralichthys dentatus*), using stable isotopes as diet tracers. Can J Fish Aquat Sci 61:2069-2084

Xia B, Gao Q-F, Dong S-L, Wang F (2013) Carbon stable isotope turnover and fractionation in grass carp *Ctenopharyngodon idella* tissues. Aquat Biol 19:207-216

Zuanon JAS, Pezzato AC, Ducatti C, Barros MM, Pezzato LE, Passos JRS (2007) Muscle delta C-13 change in Nile tilapia (*Oreochromis niloticus*) fingerlings fed lants grain-based diets. Comp Biochem Physiol A-Mol Integr 147: 761-765
